# SMLocalizer, a GPU accelerated ImageJ plugin for single molecule localization microscopy

**DOI:** 10.1093/bioinformatics/btx553

**Published:** 2017-09-04

**Authors:** Kristoffer Bernhem, Hjalmar Brismar

**Affiliations:** 1 Science for Life Laboratory, Department of Applied Physics, Royal Institute of Technology, Stockholm, Sweden; 2 Science for Life Laboratory, Department of Women’s and Children’s Health, Karolinska Institutet, Stockholm, Sweden

## Abstract

**Summary:**

SMLocalizer combines the availability of ImageJ with the power of GPU processing for fast and accurate analysis of single molecule localization microscopy data. Analysis of 2D and 3D data in multiple channels is supported.

**Availability and implementation:**

Plugin freely available for Fiji and ImageJ2.0 through https://sourceforge.net/projects/smlocalizer/. Plugin also available for continuous updates through ImageJ update system, add http://sites.imagej.net/Cellular-Biophysics-KTH/ as update site in ImageJ. Java and CUDA source code freely available on the web at https://github.com/KristofferBernhem/SMlocalizer.

**Supplementary information:**

Supplementary data are available at *Bioinformatics* online.

## 1 Introduction

Single molecule localization microscopy (SMLM) gives sub diffraction limited 2D (Betzig *et al.*, 2006; Folling *et al.*, 2008; Heilemann *et al.*, 2009; Hess *et al.*, 2006; Rust *et al.*, 2006) and 3D (Baddeley *et al.*, 2011; Huang *et al.*, 2008; Juette *et al.*, 2008; Pavani *et al.*, 2009) information. This information can be presented in the form of localization tables with coordinates and fit parameters for all identified molecules and rendered as images. The key concept in SMLM is the ability to control the state of fluorescent molecules between an ON and an OFF state in order to isolate and localize the individual fluorophore molecules, as implemented in e.g. PALM (Betzig *et al.*, 2006), fPALM (Hess *et al.*, 2006), STORM (Rust *et al.*, 2006), dSTORM (Heilemann *et al.*, 2009), GSDIM (Folling *et al.*, 2008). 3D localization can be achieved by several optical methods (Baddeley *et al.*, 2011; Huang *et al.*, 2008; Juette *et al.*, 2008; Pavani *et al.*, 2009), including controlled aberrations in the microscope beam path. All 2D methods can be analyzed using the same basic algorithms, special considerations are required for analysis of the different 3D SMLM modalities. The precision in all SMLM methods is dependent on sample preparation (Whelan and Bell, 2015), imaging conditions, optical setup (Betzig *et al.*, 2006; Heilemann *et al.*, 2009; Hess *et al.*, 2006; Rust *et al.*, 2006) as well as the parameters used for computational analysis (Sage *et al.*, 2015). Incorrect or incomplete parameters for the detection algorithms result in incorrect localizations and possibly subsequent erroneous conclusions.

## 2 Software description

Here we present SMLocalizer, a ImageJ2 (Schindelin *et al.*, 2015) plugin for SMLM image processing that is developed based on a combination of established SMLM algorithms. SMLocalizer has been developed in an effort to reduce the complexity in SMLM processing. A concern with many of the available software for SMLM processing is the dependency on sophisticated input parameter selections, often accompanied with a steep learning curve for the novice user. In SMLocalizer we have reduced this complexity by a development of iterative algorithms and reduce in most applications the requirements for user input to only two basic imaging parameters, the pixel size and the gain of the camera. For the advanced user, it is possible to manually modify all parameters in the user interface. The current version, 2.x, supports 3D SMLM through PRILM, double helix, astigmatism and biplane modalities.

### 2.1 Availability and reuse potential

SMLocalizer will run on any system capable of running ImageJ2 or Fiji (Schindelin *et al.*, 2012), see imagej.net for details on system requirements. SMLocalizer require java 8 runtime or later. GPU computing requires an NVIDIA geforce GTX 970 or later version. Older cards may work if sufficient memory is available.

By using the ImageJ interface for data input and output, SMLocalizer is independent on the source file format and compatible with all commercial and custom built SMLM systems currently available.

A detailed user manual detailing how to operate the software as well as details concerning algorithms is available online, see Supplementary Material and https://sourceforge.net/projects/smlocalizer/. The plugin is available for continuous updates through ImageJs update system, add http://sites.imagej.net/Cellular-Biophysics-KTH/ as an update site in ImageJ/Fiji to keep the plugin up to date.

### 2.2 Comparison with other SMLM software

**Fig. 1:**
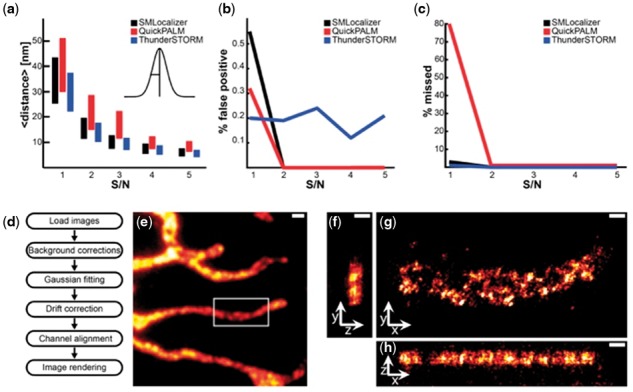
Quality control and comparison, architecture and 3 D example. Comparison of SMLocalizer, QuickPALM and ThunderSTORM using five datasets with increasing peak signal to peak noise ratio (S/N). (**a**) Mean distance from ground truth. Center of bars represent the mean distance and height of bars represent standard deviation of the sample. (**b**) False positive localizations. (**c**) Total missed true localizations. (**d**) The basic analysis workflow of SMLocalizer. (**e**) Widefield image of U2OS cells stained for mitochondrial Mitofilin with an Alexa-Fluor647 secondary antibody. Scale bar is 1 µm. (**f–h**) ZY (**f**), XY (**g**) and XZ (**h**) projection of SMLocalizer analyzed and rendered results of 3 D [PRILM (Baddeley *et al.*, 2011)] imaging of the sample in e). Image is rendered with intensity representing binned localization densities that has subsequently been filtered using a 10 nm σ Gaussian. Scale bars are 250 nm (see Supplementary Methods)

During development, all functions were evaluated against ground-truth based synthetic datasets. A final comparison of SMLocalizer against QuickPALM (Henriques *et al.*, 2010) and ThunderSTORM (Ovesný *et al.*, 2014) was performed using five ground-truth datasets with random, known, activation of a single particle with different signal to noise ratios (Fig. 1a–c). In summary, SMLocalizer performs as well as other available software but required less user input and performed faster (2–15 fold). A detailed discussion of the comparison and quality control is available in the Supplementary Material.

### 2.3 Architecture

A schematic representation of the processing steps of SMLocalizer can be found in Figure 1d. SMLocalizer uses a graphical interface for all input from the user. The first step in processing SMLM data through SMLocalizer is correction of background. By removal of the time median of each pixel, static elements are removed and only blinking events retained (Hoogendoorn *et al.*, 2014). Shot-noise is removed from the static element corrected images using a bicubic b-spline kernel (Unser, 1999). Background corrected image regions (500 nm wide for 2D) are fitted against a 2D Gaussian by minimizing the least square errors of fit generating a raw localization table. For 3D data the fits are mapped against a calibrated lookup table to yield 3D localizations. Optional drift correction is performed through autocorrelation and channels are aligned by maximizing channel correlation.

After processing all data is available as a standard result table in ImageJ that can be exported for further analysis in other software. In a final step images can be rendered and visualized directly in ImageJ and be subjected to all image processing functions available in ImageJ.

## Supplementary Material

Supplementary User Manual

Supplementary Methods
