# Supplementary material for: SMLocalizer, a GPU accelerated ImageJ plugin for single molecule localization microscopy
Source: Bioinformatics. 2017 Sep 4;34(1):137–8. doi: 10.1093/bioinformatics/btx553 (PMC5870682; doi:10.1093/bioinformatics/btx553)
Supplement: Supplementary User Manual [file smlocalizer_user_manual_btx553.pdf]

SMLocalizer user manual

Version 2.0

2017 – Kristoffer Bernhem, Hjalmar Brismar

1 CONTENTS

2 Setup..3
2.1 Requirments....................................3
2.1.1 ImageJ 3
2.1.2 OS and hardware requirements3
2.1.3 GPU acceleration....................3
2.2 Installing SMLocalizer....................4
3 Processing SMLM data 5
3.1 Processing.......................................5
3.2 Loading images and settings 5
3.3 Calibration.......................................6
3.4 Background correction 7
3.5 Fitting 8
3.5.1 Modality selection...................9
3.5.2 Image pixel size.......................9
3.5.3 Total gain................................9
3.5.4 Minimal signal.........................9
3.6 Parameter selection........................10
3.6.1 Clean table.............................10
3.6.2 Photon count..........................10
3.6.3 Sigma x y...............................11
3.6.4 R^2 11
3.6.5 Precision x y 11
3.6.6 Precision z 11
3.6.7 Frame....................................11
3.6.8 Z 11
3.7 Drift correction and channel alignment..11
3.7.1 Max drift 12
3.7.2 Number of bins 12
3.8 Image rendering 13
3.9 Cluster analysis 13
3.10 Multichannel images..................... 14
3.11 Parallel vs GPU computation........ 14
3.12 Subsequent analysis in Matlab...... 14
4 Tutorial – SMLM challenge 16
4.1 2D 16
4.2 3D Double Helix........................... 19
4.2.1 Calibration 19
4.3 Mitofilin PRILM........................... 24
4.4 2D Gold bead tutorial – Drift correction 27
5 Architecture and algorithms.................. 28
5.1 Background correction.................. 28
5.1.1 Static events removal 28
5.1.2 Shot noise............................. 28
5.2 Fitting... 29
5.2.1 2D fitting.............................. 29
5.2.2 3D fitting.............................. 30
5.2.3 Z precision estimate for PRILM and double helix................................... 35
5.3 Drift correction and channel alignment 36
5.4 Image rendering 36
5.5 Cluster analysis 36
6 References.. 37

2 SETUP

2.1 REQUIRMENTS

2.1.1 ImageJ

SMLocalizer require ImageJ2.0[1] (http://imagej.net/) or Fiji[2] (https://fiji.sc/) to function. SMLocalizer will require writing privileges to the preference file ImageJ or Fiji.

2.1.2 OS and hardware requirements

SMLocalizer will run on any system capable of running ImageJ or Fiji. At time of writing this includes (taken from http://imagej.net/Downloads) :

ImageJ will run on any system that has a Java 8 (or later) runtime installed. This includes, but is not limited to:

1. Windows XP, Vista, 7 or 8 with Java installed from java.com
2. Mac OS X 10.8 "Mountain Lion" or later with Java installed from java.com
3. Ubuntu Linux 12.04 LTS or later with OpenJDK 8 installed

SMLM images are typically large, as such, SMLocalizer requires that the java heap space memory be set high enough for the files the user intends to load. Typical values should be 3x the raw data file size. Java heap space memory is set in */Edit/Options/Memory & Threads*.

2.1.3 GPU acceleration

SMLocalizer by default runs all processes on the system available CPU cores. If a compatible NVIDIA card is available, the user can select to transfer functions to the GPU instead. For GPU accelerated computation SMLocalizer 2.x.x requires a NVIDIA GeForce gtx970 or later graphics card. Older version may work but none have been tested. The main limitation is memory available, with the current code expecting 4 GB of memory available. Ensure that the latest drivers for the graphics card are installed. For windows, timeout can be an issue for larger data files, this can be fixed through changing the graphic device timeout to 10+ seconds, see http://answers.microsoft.com for details on this.

The OS specific CUDA archives needs to be places in \plugins\jars directory of the main ImageJ directory (or into \jars for Fiji). Download the Fiji or ImageJ specific .rar and extract it into the main directory.

2.2 INSTALLING SMLOCALIZER

In ImageJ or Fiji, go to the help menu and select *Update* (see fig 1). This will after a checking of your current plugins take you to the updater window.

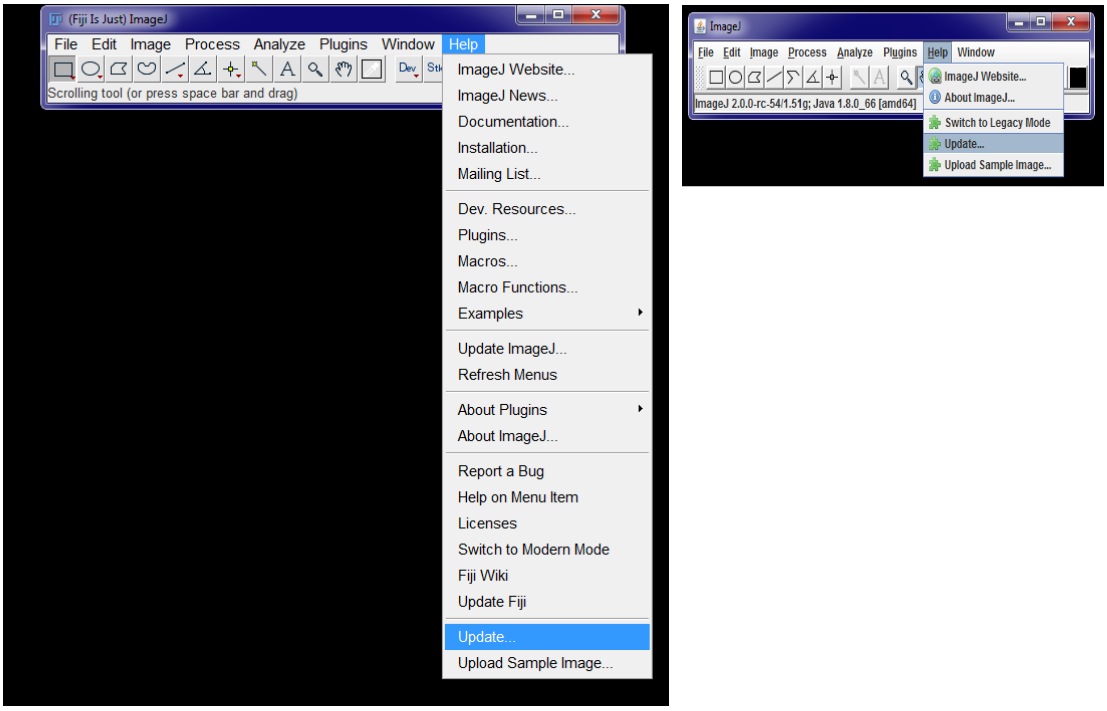

Figure 1 Fiji and ImageJ update.

Select *Manage update sites* and *Add update site*. A new line will be added to the list of update sites (see fig 2). Replace the name *New* with *SMLocalizer* and add the URL: http://sites.imagej.net/Cellular-Biophysics-KTH/. ImageJ will now keep your SMLocalizer up to date.

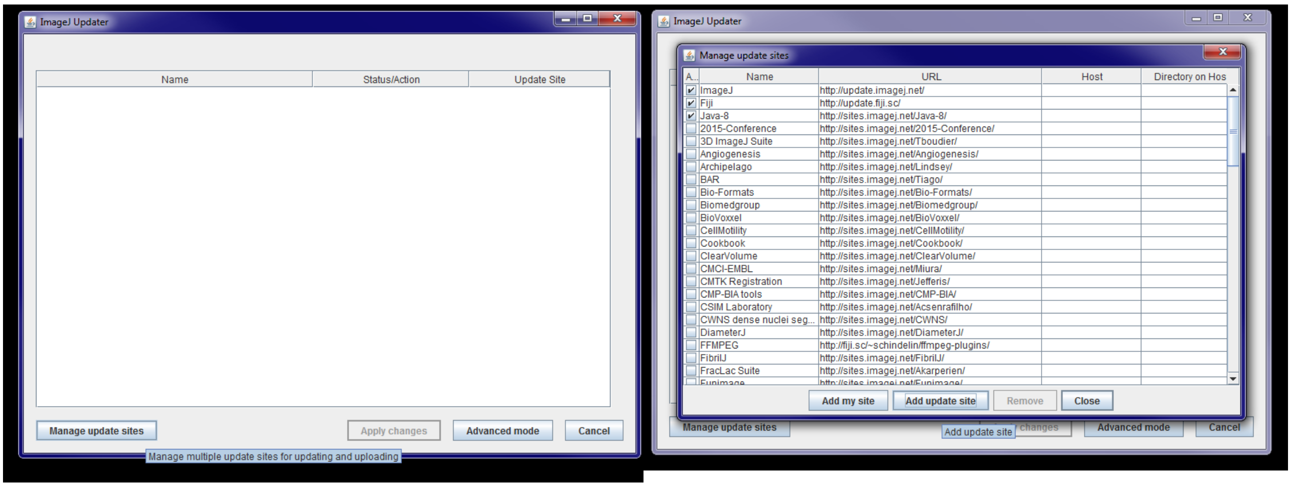

Figure 2 Adding SMLocalizer to update sites

3 PROCESSING SMLM DATA

The following section aim to explain the details required to properly use SMLocalizer. For a more detailed description of the algorithms, see section 5.

As ImageJ does not have a clear way to display processing progress, the keys used for the different algorithms will remain depressed whilst their respective algorithms are running.

3.1 PROCESSING

SMLocalizer can fit one-color 2D experiments without calibrations by:

1. Loading the data set into ImageJ.
2. Run SMLocalizer from */plugins/* menu.
3. Setting the correct *image pixel size*.
4. Setting the correct *total gain*. This value is camera manufacturer dependent. If left unmodified photon count will not yield correct values, nor precision estimates. All other parameters will be correctly fitted despite gain being incorrectly set.
5. OPTIONAL:
    a. Setting the *minimal signal* that should be considered of interest. Take the center pixel value and reduce this number by the frame mean background value. Set minimal signal to this value. If the checkbox for minimal signal is not checked the software will automatically determine regions of interest based on image intensity distributions.
    b. If dataset is > 1000 frames, leave *filter width* at 101. If smaller, reduce filter width value.
6. Press *Process*.

Once all settings have been determined the *Process* button will sequentially do:

1. Background correction.
2. Fitting of selected regions selected based on *Minimal signal*.
3. Filtering of fits based on selected parameter ranges.
4. Drift correction (if selected).
5. Channel alignment (if selected and a multichannel image stack is loaded).
6. Rendering of results (if selected).
7. Cluster analysis and rendering of results (if selected).

Each of these steps can be performed manually by the user through calling the individual algorithms. See sections 3.4 through 3.9 for details.

3.2 LOADING IMAGES AND SETTINGS

Loading images for processing by SMLocalizer is done through the main ImageJ or Fiji interface. Some image formats lack compression and require additional space. A suggestion for performance optimization is to resave these as .tif stacks before proceeding.

Once installed and an image stack has been loaded, the plugin can be started. In the *Plugins* menu, select *SMLocalizer* to start the main plugin. Upon startup SMLocalizer will load the settings last stored (or default if no new settings have been stored). New settings can be stored or loaded using the two buttons in the bottom right corner (see fig 3).

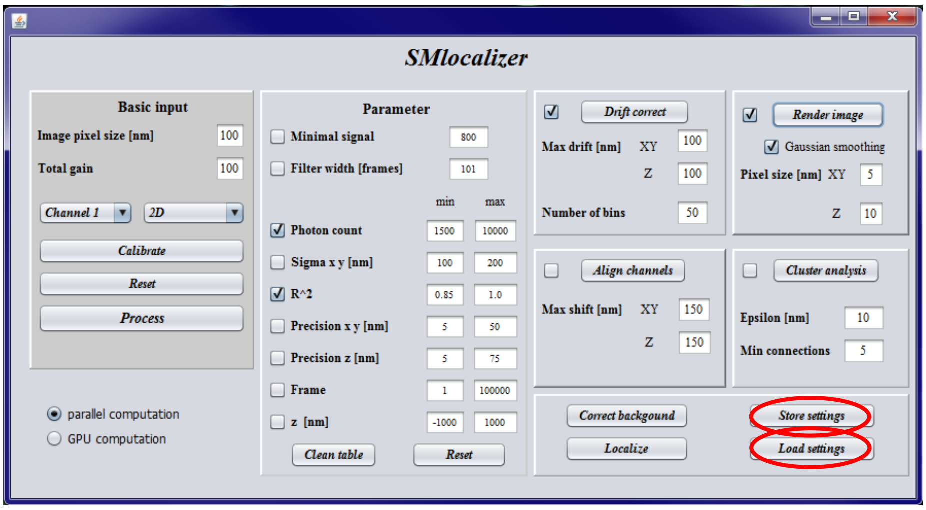

Figure 3 SMLocalizer graphical interface with relevant buttons for settings storing and loading.

3.3 CALIBRATION

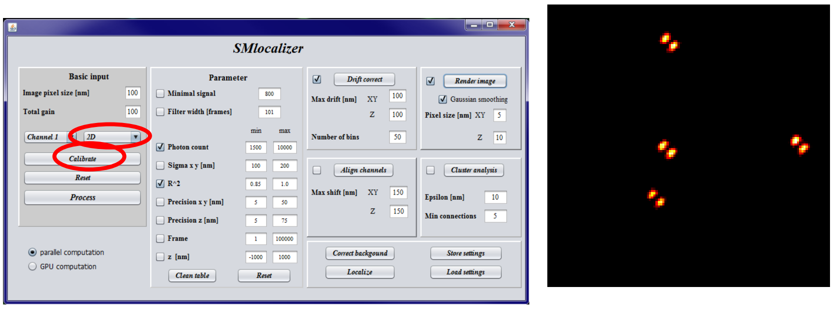

Figure 4 Relevant button and menu to calibrate 2D or 3D fits. Right panel example of central frame from 3D PRILM calibration stack.

SMLocalizer require bead images for calibration of 3D fits and can use multispectral beads for calibration of channel offset in x-y (2D) or x-y-z (3D). For 2D one-color data calibration is not required. For 2D multicolor data SMLocalizer will run but will not compensate for spectral shift between channels without calibration.

For 3D fit calibration image stacks with known z-step size with the same channel setup as for the intended experiment using sub 100 nm beads will need to be acquired. The beads need to emit light in wavelengths covering all filters that will be used in the experiments that will be fitted. So if an experiment calls for the use of the use of Atto-488 and AlexaFluor-647 as STORM dyes a bead emitting photons in the 510-525 and 650-670 nm range is required. Center focus on the beads and include in 10-20 nm steps 1 µm down and 1 µm up from this imaging plane in the image stack, generating a 100-200 frame image with two channels in the dye example above. Ensure that the order of filter imaging is the same as for the experiment, so if Atto-488 will be imaged first, place it first in the calibration experiment as well. 10-20 well-separated beads should be included in the image stack for best results. Also, ensure that you have good signal through the entire stack.

Once the stack has been loaded into ImageJ, select the correct 3D modality (or single slice multi-channel image for 2D calibration) and press *Calibrate* (see fig 4). For 3D modalities, the software will

ask you for the z-step size in nm. Once you press *OK*, the calibration will commence. Once completed you will be presented with a list of fitted data points in a result table. What is common is that the very edge of the calibration file is a more uncertain then the more central parts, and subsequently any errors will be larger at the edges of the calibration. The calibration algorithms will generate a lookup table for the modality for z fitting and a series of offset values for x-y-z against the first channel for multichannel images. This offset will be applied to all fitted 3D data before being presented to the user.

As small shifts in system alignment will affect the performance, new calibration files should be generated often, preferably weekly for good performance.

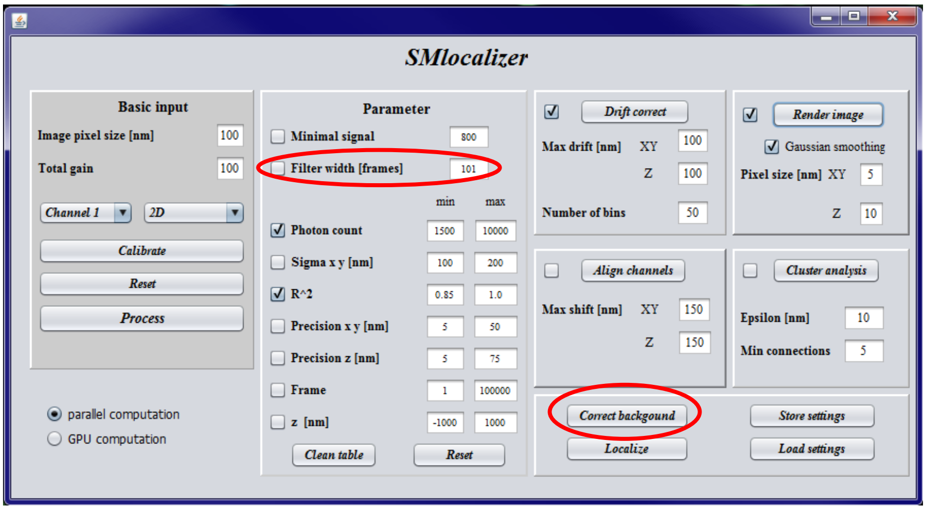

Figure 5 Relevant buttons and fields for background correction.

3.4 BACKGROUND CORRECTION

SMLocalizer performs background correction based on the work of Hoogendoorn el al[3]. In short, static signal (for longer than half the filter width (see fig 5) from *Basic input*) is removed and transient increases remain (an example before and after image can be seen in fig 6). Background correction can be done through the *Process* button that will start with background correction or through *Correct background* which will background correct and replace the current open image stack (see fig 6). SMLocalizer will use a default 101 frame width for background corrections if the checkbox is not checked and the value for the channel altered.

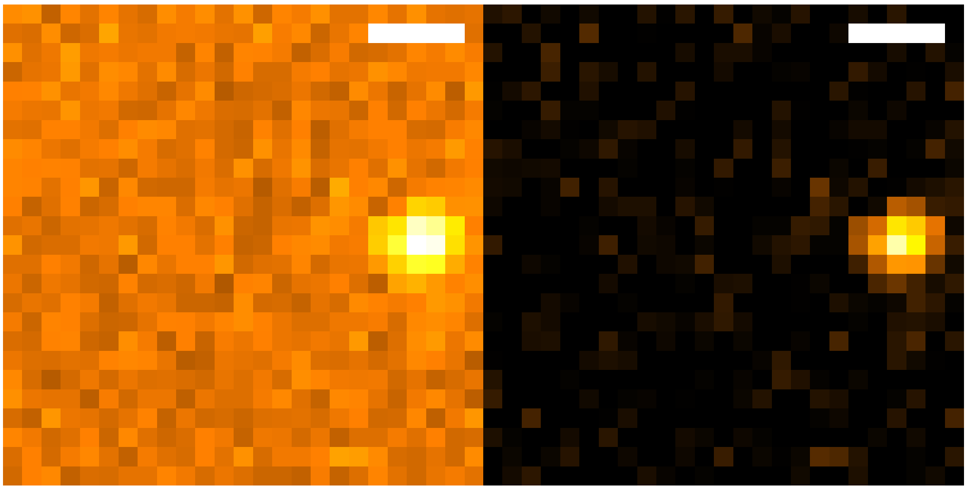

Figure 6 With same color map, example of before and after background filtering. 500 nm scalebar.

3.5 FITTING

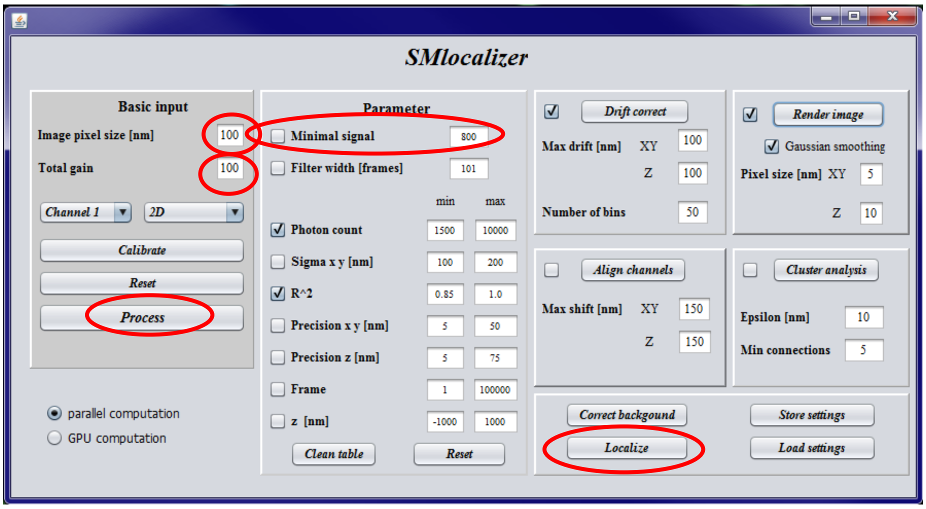

Figure 7 Relevant buttons and fields for Gaussian fitting.

SMLocalizer performs 2D Gaussian and 3D multi-modality fitting on regions extracted from the open image stack through minimizing least square errors of fit. Two settings (*Pixel size* and *Total gain*) needs to be looked over for the Gaussian fitting with an additional parameter that can be modified for experienced users, *Minimal signal* (see fig 7). This will override SMLocalizers standard region identification to only include areas with center pixels stronger then *Minimal signal* (after background correction).

Results

File Edit Font Results

	x0	y0	z0	frame	channel	sigma_x	sigma_y	precision_x	precision_y	precision_z	r_square	photons
1	3783.057115464	2259.143579170	0	1	1	115.186545448	101.053002088	2.257258326	1.980289707	0	0.994752274	2604
2	2316.661985122	4173.238120903	0	1	1	121.667500000	113.332500000	2.096158309	1.952558091	0	0.991146681	3369
3	3786.744202960	2262.943738310	0	2	1	130.000000000	115.701522772	2.559372501	2.277871505	0	0.984856335	2580
4	2314.030388262	4176.607343392	0	2	1	119.405226448	111.137054102	2.060549209	1.917867214	0	0.994414895	3358
5	3785.130392564	2260.122463656	0	3	1	118.692138928	101.158299283	2.328192128	1.984259094	0	0.993578005	2599
6	2314.858477819	4178.772169178	0	3	1	121.667500000	113.332500000	2.081078944	1.938511759	0	0.991149734	3418
7	3785.524786974	2257.164367826	0	4	1	115.047931024	100.205639493	2.267201897	1.974711009	0	0.994677870	2575
8	2319.221965544	4177.692828311	0	4	1	130.000000000	121.667500000	2.243380840	2.099588757	0	0.982967786	3358
9	3788.982428265	2256.061357764	0	5	1	114.498141531	101.273081102	2.262084765	2.000803601	0	0.995530617	2562
10	2324.973939099	4177.815433707	0	5	1	130.000000000	121.667500000	2.247064280	2.103036102	0	0.979599458	3347
11	3790.273691414	2261.264400554	0	6	1	130.000000000	117.652222093	2.582498316	2.337205119	0	0.978805768	2534
12	2318.387524413	4172.214781168	0	6	1	121.667500000	113.332500000	2.102722005	1.958672132	0	0.991057930	3348

Figure 8 Example result table after fitting of particles.

3.5.1 Modality selection

SMLocalizer can fit both 2D and 3D SMLM data. In the dropdown menu at the bottom half of *Basic input* (see fig 7), the user can select between 2D and different 3D modalities. In order for fit 3D data, a calibration needs to be generated for that modality. See *Calibration* (3.2) for details concerning how to generate calibrations. For 3D, select the correct modality from the list:

- PRILM[4]
- Biplane[5]
- Double helix[6]
- Astigmatism[7]

SMLocalizer will proceed to use the calibration for that modality and fit the 3D data, returning x-y-z coordinates for all events that fall within the calibration range. 2D and 3D data are then handled in the same way with regards to parameter filtering, image rendering, channel alignment and drift correction. For details concerning how 3D modalities are processed, see section 5.2. For Biplane data the input data needs to be stitched so that both planes are in a single image next to each other.

3.5.2 Image pixel size

User set parameter that is camera dependent. Change this value to the value that your system uses.

3.5.3 Total gain

This is the total conversion rate from a photon being read by the camera to the resulting pixel intensity. Camera manufacturers provide information on how this number is obtained.

3.5.4 Minimal signal

Minimal intensity of center pixel of a region. Pixels below this value will not be considered a possible particle. By default this is not used but rather particles will be located automatically if the center pixel is frame mean + 0.7σ or stronger.

3.6 PARAMETER SELECTION

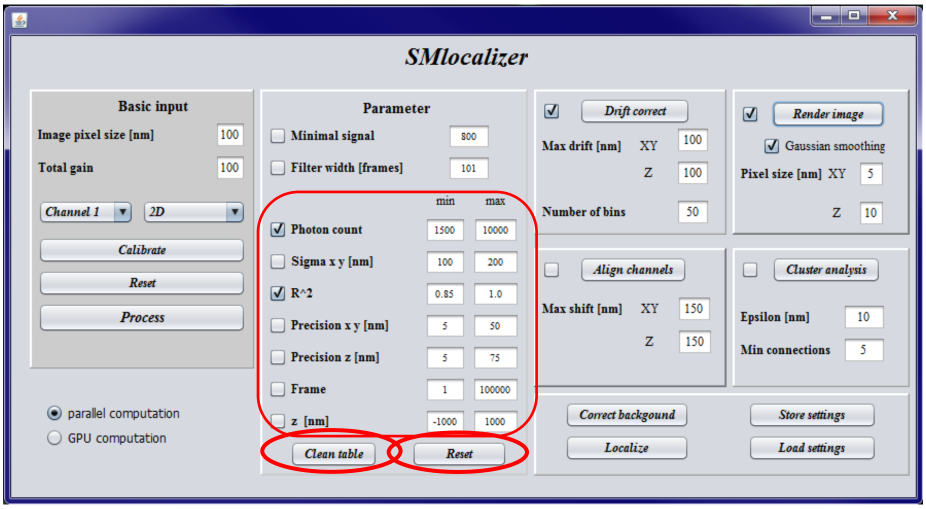

Figure 9 Relevant buttons and fields for parameter range settings.

Parameter range (see fig 9) is used to select which fitted particles should be included in downstream algorithms. By selecting a parameter type, that type will be used and only particles with values between min and max will be included. Setting parameter range is non-destructive, that is, particles outside of the selected range are not removed but only excluded. An example list of what the parameter distribution can look like after fitting can be found in Figure 8.

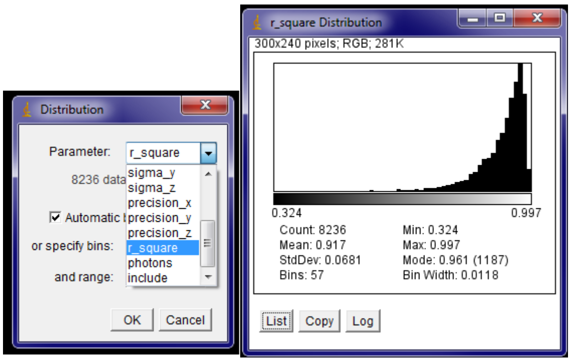

Figure 10 Plotting of parameter distributions

The distribution of each parameter can be obtained from the result table. Select the *Result* menu and *Distribution*. Select the parameter of interest to plot the selected distribution (see Figure 10).

3.6.1 Clean table

By clicking *Clean table* SMLocalizer will remove particles that fall outside the selected parameters range. This is destructive.

3.6.2 Photon count

This is the number of photons SMLocalizer has calculated a particle has emitted. For PALM it is reasonable to require 100+ photons per particle whilst for STORM imaging 500+ photons should be required.

3.6.3 Sigma x y

This is the sigma in x and y for the Gaussian fit. Sigma of the Gaussian fit relates to the full width at half maximum (FWHM) as $FWHM = 2\sqrt{2ln2}\sigma$ (wide field). The width of an in focus emitter will change depending on the optics and filters used but is typically in the range of 100-200 nm. Out of focus emitters will have a broadening of their PSF in 2D which will be reflected by larger values of sigma.

3.6.4 R^2

This is the goodness of fit for a given particle. A value of 1.0 is a perfect fit and 0.0 would indicate no correlation between fit and real data. With decreased R^2 values come an uncertainty in the localization. In our experience values above 0.85 is a good compromise.

3.6.5 Precision x y

This is the precision of the Gaussian fit in x and y. Dependent on sigma x y and photon count. Precision of the fit scales with the square root of the number of photons. This will be highly probe dependent but reasonable values typically fall between 5 – 50 nm.

3.6.6 Precision z

This is the precision of the Gaussian fit in z. Precision of the fit scales with the square root of the number of photons for astigmatism and biplane modalities. For PRILM and Double Helix modalities, the error in x-y determination is used to calculate the precision in z. This will be highly probe dependent but reasonable values typically fall between 5 – 75 nm. If the precision calculations for PRILM or Double Helix falls outside the calibration range a value of 1000 will be reported.

3.6.7 Frame

Which frames to include. Discarding early portions with to high density of events can improve upon results.

3.6.8 Z

Which z coordinates to include. SMLocalizer will during 3D fitting set z = 0 at the plane of focus with z decreasing towards the beginning of the calibration stack and increasing towards the end of the calibration stack. Any 3D modality will be more accurate in the region surrounding z = 0.

3.7 Drift correction and channel alignment

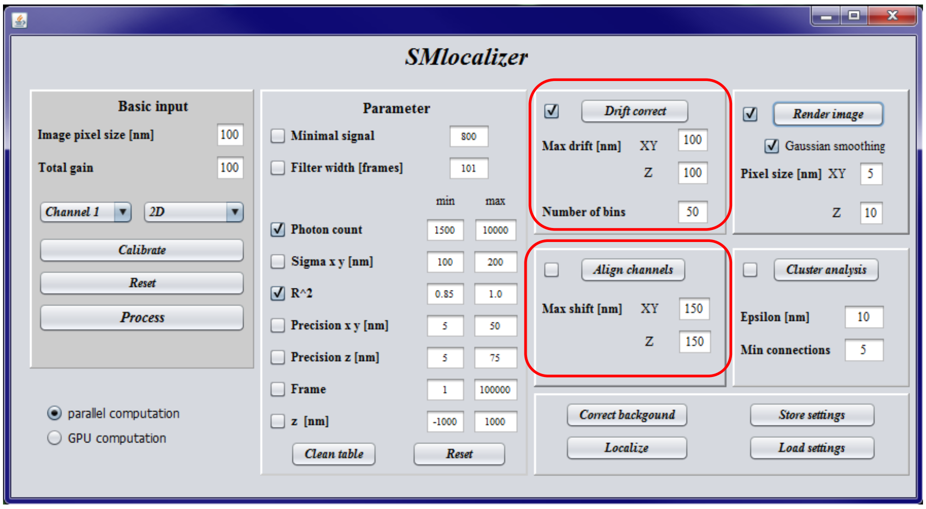

Figure 11 Buttons and relevant fields for drift correction and channel alignment.

SMLocalizer will perform drift correction on the fitted particles during processing if the checkbox is checked (see fig 11). By clicking the *Drift correct* button (see fig 11), a result table of fitted particles

can be drift corrected outside of the main sequence of processing performed by the *Process* button (see fig 11). Drift correction is performed by maximizing the correlation between two adjacent bins of particles, separated based on frame number. Drift correction is done on each channel separately. Drift correction is prone to artefact introduction if too few particles are included.

Alignment of channels is performed in the same manner, accounting for drift between start of channel acquisition and chromatic shifts.

3.7.1 Max drift

The maximum drift from one bin to the next in X/Y and Z separately. Smaller allowed drifts will reduce computational cost at the risk of not finding the correct drift.

3.7.2 Number of bins

The number of bins (in time) the fitted particles should be divided into.

3.8 Image Rendering

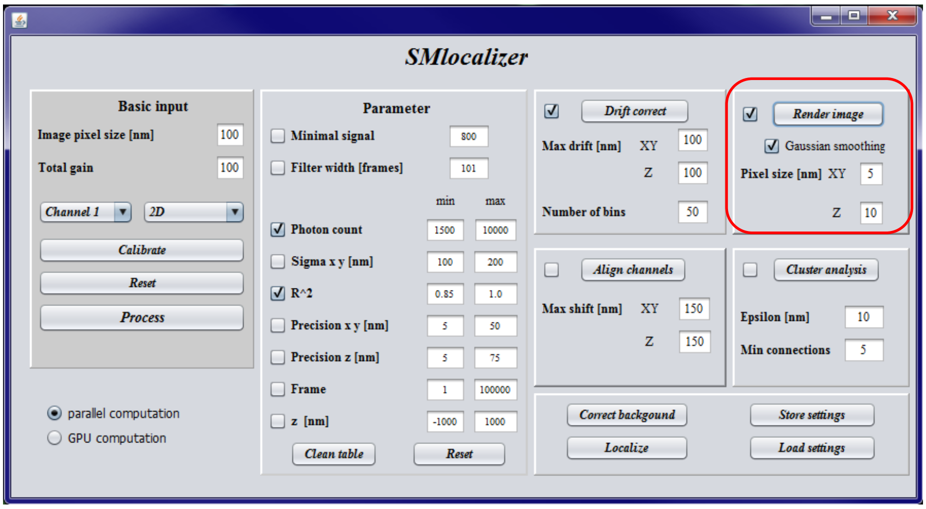

Figure 12 Buttons and fields relevant for image rendering.

SMLocalizer will, if the checkbox for *Render image* is selected (see fig 12), perform this action during *Process*. *Render image* uses the particles within the selected parameter range to render an image with a pixel size set by the user. For 3D data an image stack will be created with the voxel size chosen by the user (see fig 12). Default is that each particle adds a value of one to the image, allowing for counting particles in the image. All image editing ImageJ or Fiji is capable of performing can be performed on these result images. *Gaussian smoothing* multiplies the image values and applies a two pixel wide Gaussian filter.

3.9 Cluster Analysis

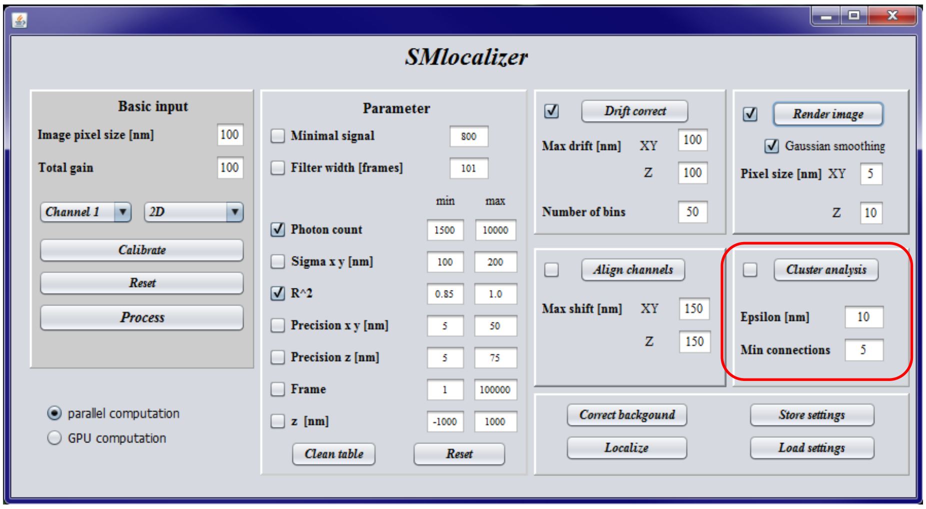

Figure 13 Buttons and fields relevant for cluster analysis.

SMLocalizer can perform 2D cluster analysis on the localized particles through the DBSCAN[8] algorithm. A results image of the clusters found will be generated based on the pixel size set in *Render image* (see 3.8). Particles will be considered to be part of a cluster if they have *Min connections* neighbors within *Epsilon* (see fig 13).

3.10 MULTICHANNEL IMAGES

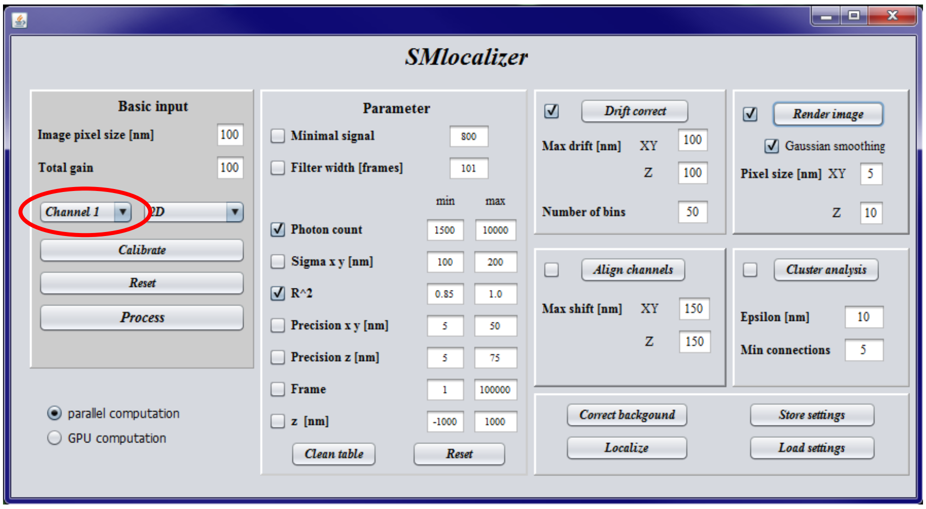

Figure 14 Multichannel selection.

SMLocalizer can analyze multichannel (but not currently multi frame and slice) images. At the bottom part of the *Basic input* tab (see fig 14), a list of channels is available. Default is a single channel but more can be added in the list. Each channel has its own settings for all fields and checkboxes that needs to be set.

3.11 PARALLEL VS GPU COMPUTATION

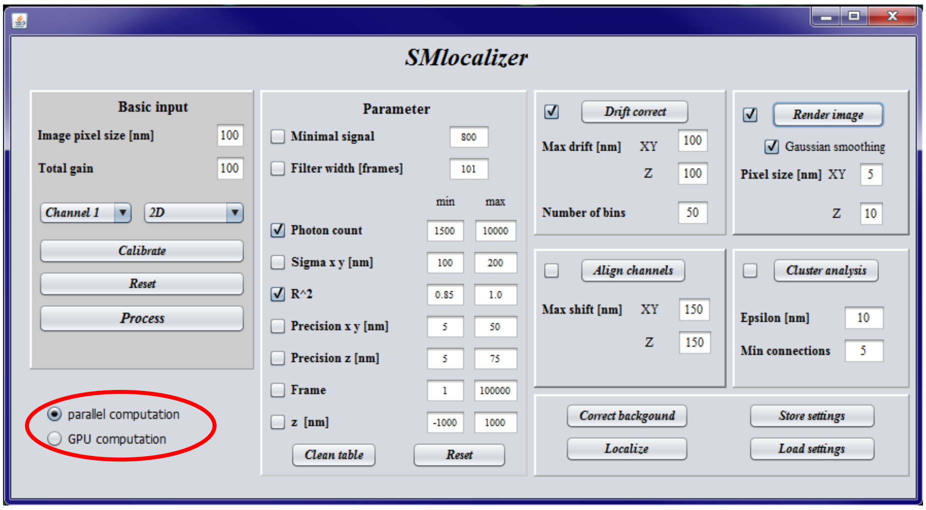

Figure 15 Selection of parallel vs GPU accelerated computation.

SMLocalizer can on CUDA capable systems (see 2.1.3) perform the computationally intensive calculations on a GPU. The default alternative is CPU based parallel computing (see fig 15).

3.12 SUBSEQUENT ANALYSIS IN MATLAB

Once a results table has been obtained from SMLocalizer the data can be transferred to any software that accepts .tif or tab separated tables as input. For Matlab a short function is available online on https://sourceforge.net/projects/smlocalizer/ that will translate the results table into a struct in Matlab containing vectors with all data.

To transfer SMLM data processed in SMLocalizer to Matlab, click *File/SaveAs* in the results table and store the file, adding *.txt* at the end of the file name. Once this has been done, download the matlab

function *LoadSMLocalizer.m* from Sourceforge, start Matlab and add *LoadSMLocalizer.m* to the path (through the *Set Path* button or by copying the file to the folder containing your other custom Matlab functions). To load the data, use the call:

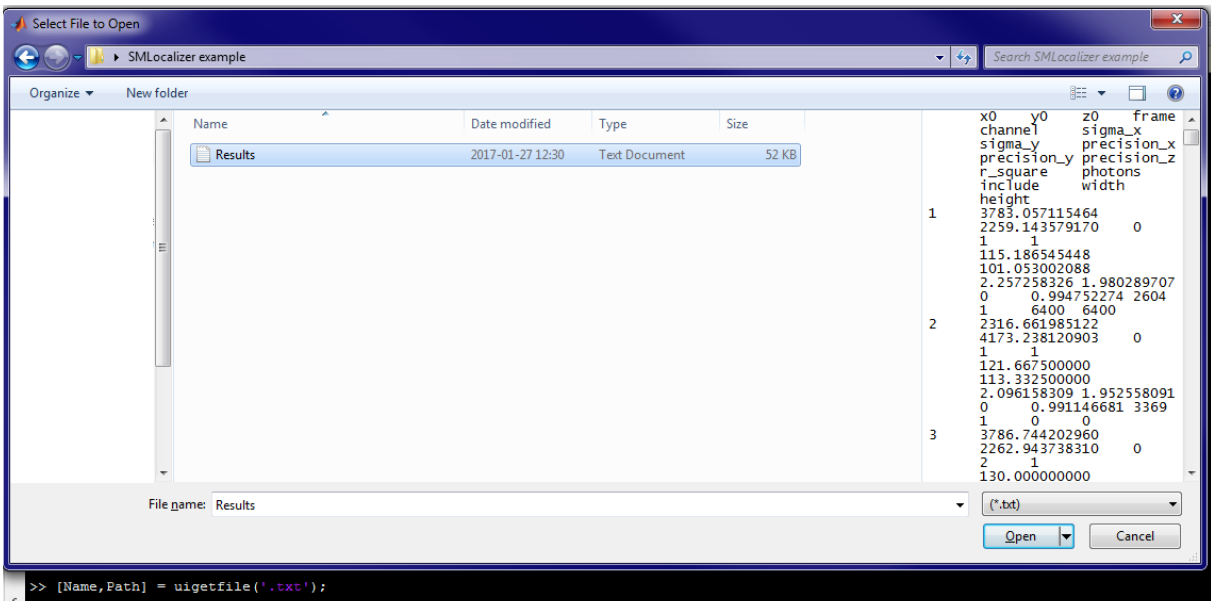

Figure 16 GUI shown after the uigetfile('.txt') command has been called in matlab.

[Name,Path] = uigetfile('.txt'); (see figure 16)

Select the file of interest and click ok. Proceed with the next function call:

S=LoadSMLocalizer([Path,Name]); (see figure 17)

S will now contain all data from the results table, available for further Matlab based analysis.

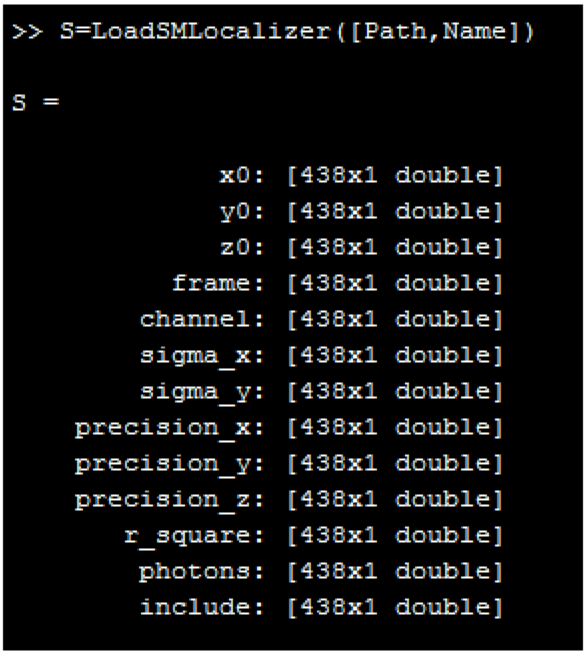

Figure 17 Output after calling LoadSMLocalizer with Path and Name from uigetfile call.

4 TUTORIAL – SMLM CHALLENGE

4.1 2D

This tutorial will give an example of how SMLocalizer can be used. This example uses the *sequence-as-stack-MT0.N1.LD-2D-Exp* dataset from the 2016 SMLM challenge (http://bigwww.epfl.ch/smlm/challenge2016/index.html). To download the dataset, go to http://bigwww.epfl.ch/smlm/challenge2016/datasets/MT0.N1.LD/Data/data.html and download *MT0.N1.LD-2D-Exp-as-stack*. A 3D example follows this 2D example.

Start by downloading the dataset and install SMLocalizer into ImageJ/Fiji (this tutorial uses Fiji).

All color maps have been changed to *Red hot*.

1. Load the dataset into Fiji.
2. Zoom in image to 800 %.
3. Go to *Image/Adjust/Brightness / Contrast* and press *Reset* in the B&C panel now displayed. Next zoom in on the image to a 800% zoom (see fig 18).

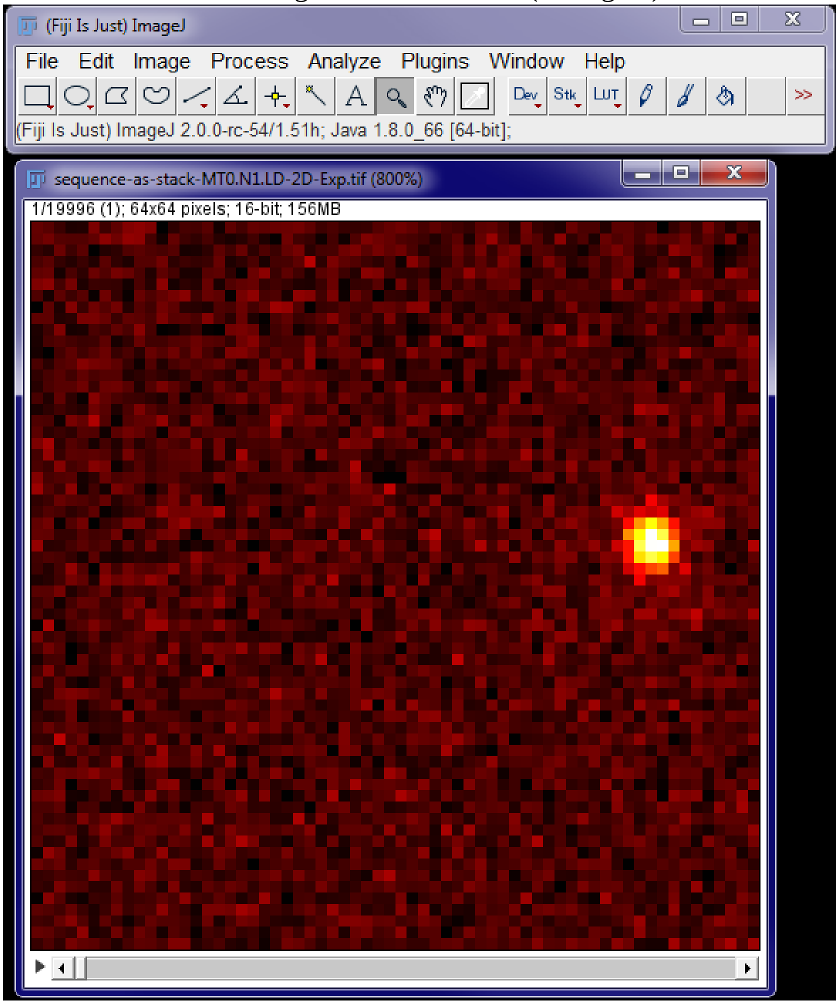

Figure 18 Initialize Fiji and load the imagestack.

4. Start SMLocalizer.

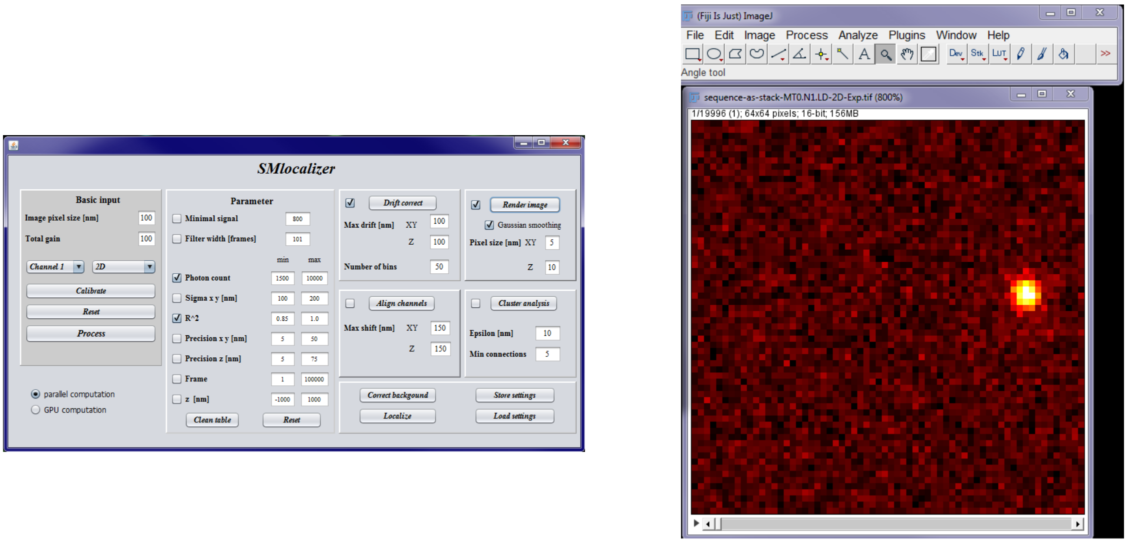

Figure 19 Initialize SMLocalizer.

5. At this point the user can either activate *Gaussian smoothing* in the *Render image* tab and press *Process* or go through the steps detailed below (see fig 19).
6. Hit *Correct background* key (see fig 20).

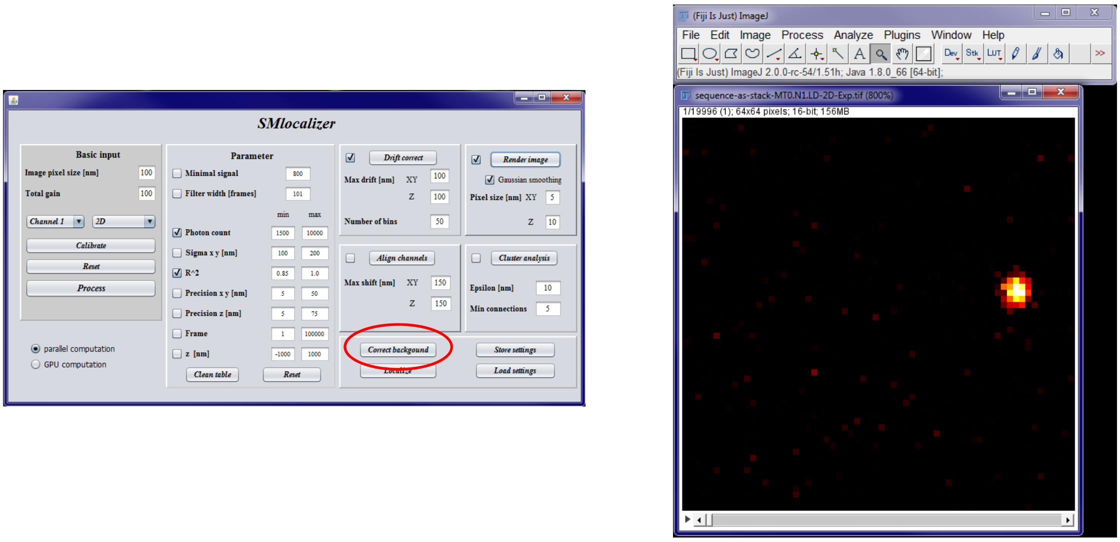

Figure 20 SMLocalizer after background correction.

Press *Localize* (see fig 21 for output).

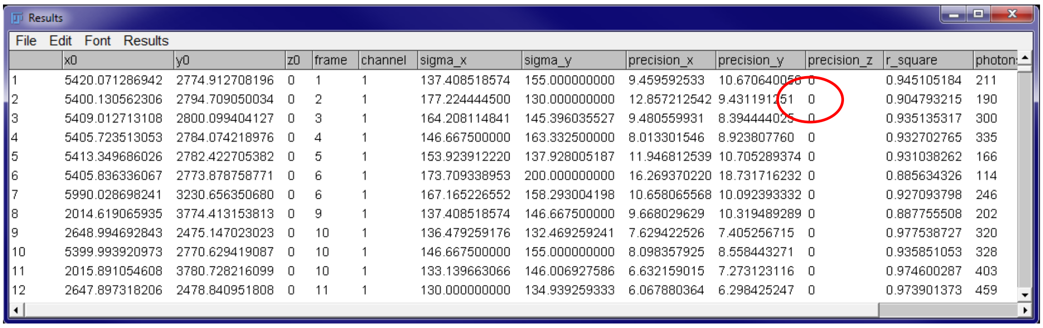

Results

File Edit Font Results

	x0	y0	z0	frame	channel	sigma_x	sigma_y	precision_x	precision_y	precision_z	r_square	photon
1	5420.071286942	2774.912708196	0	1	1	137.408518574	155.000000000	9.459592533	10.670640058	0	0.945105184	211
2	5400.130562306	2794.709050034	0	2	1	177.224444500	130.000000000	12.857212542	9.431191251	0	0.904793215	190
3	5409.012713108	2800.099404127	0	3	1	164.208114841	145.396035527	9.480559931	8.394444025	0	0.935135317	300
4	5405.723513053	2784.074218976	0	4	1	146.667500000	163.332500000	8.013301546	8.923807760	0	0.932702765	335
5	5413.349686026	2782.422705382	0	5	1	153.923912220	137.928005187	11.946812539	10.705289374	0	0.931038262	166
6	5405.836336067	2773.878758771	0	6	1	173.709338953	200.000000000	16.269370220	18.731716232	0	0.885634326	114
7	5990.028698241	3230.656350680	0	6	1	167.165226552	158.293004198	10.658065568	10.092393332	0	0.927093798	246
8	2014.619065935	3774.413153813	0	9	1	137.408518574	146.667500000	9.668029629	10.319489289	0	0.887755508	202
9	2648.994692843	2475.147023023	0	10	1	136.479259176	132.469259241	7.629422526	7.405256715	0	0.977538727	320
10	5399.993920973	2770.629419087	0	10	1	146.667500000	155.000000000	8.098357925	8.558443271	0	0.935851053	328
11	2015.891054608	3780.728216099	0	10	1	133.139663066	146.006927586	6.632159015	7.273123116	0	0.974600287	403
12	2647.897318206	2478.840951808	0	11	1	130.000000000	134.939259333	6.067880364	6.298425247	0	0.973901373	459

Figure 21 After events has been localized a result table is displayed.

7. For this set there are not enough events to do any meaningful drift correction. Pressing *Drift correct* will tell the user that no correction could be done under these settings. We do not suggest reducing the demands as artifacts are likely to appear.
8. Instead, render the result table using *Gaussian smoothing* and a *R^2* range of 0.85-1.0. To do activate *Gaussian* smoothing in the Render tab, activate *R*^2 selection and set its range to 0.85 – 1.0 in the parameter selection tab and finally press *Render* Image (see fig 22).

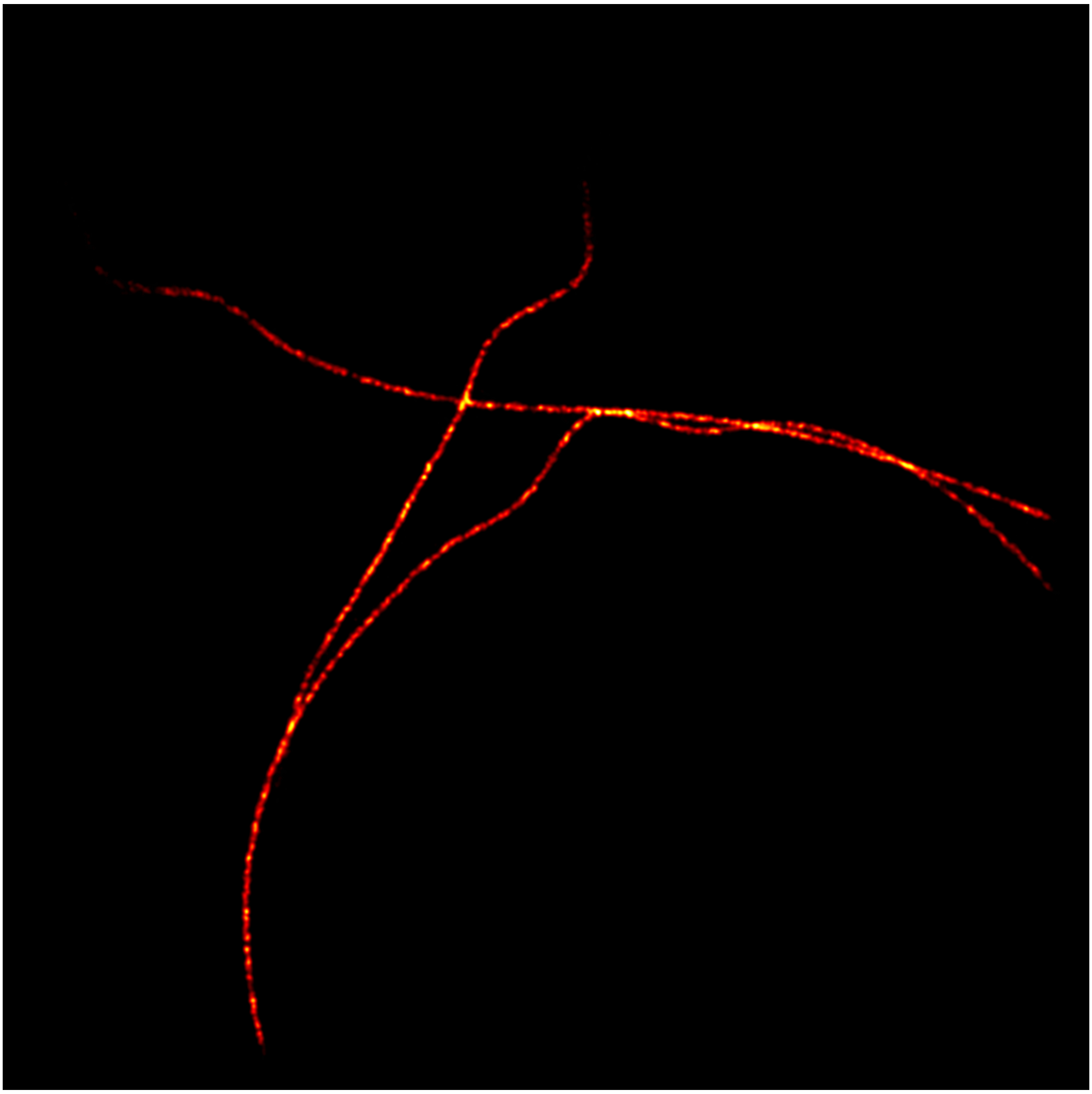

Figure 22 Final rendered results

4.2 3D DOUBLE HELIX

This tutorial will give an example of how SMLocalizer can be used for a 3D dataset. This example uses the sequence-as-stack-MT0.N1.LD-DH-Exp and the sequence-as-stack-Beads-DH-Exp datasets from the 2016 SMLM challenge (http://bigwww.epfl.ch/smlm/challenge2016/index.html). The *sequence-as-stack-Beads-DH-Exp* is used for 3D calibration and applied to the *sequence-as-stack-MT0.N1.LD-DH-Exp* dataset. In order to download the datasets, go to http://bigwww.epfl.ch/smlm/challenge2016/datasets/MT0.N1.LD/Data/data.html and download *MT0.N1.LD-DH-Exp-as-stack* and then go to http://bigwww.epfl.ch/smlm/challenge2016/datasets/Beads/Data/data.html and download *z-stack-Beads-DH-Exp-as-stack.zip*. All image displays use *red hot* lookup table for 2D.

4.2.1 Calibration

Start by loading the calibration stack into ImageJ and start SMLocalizer from the /plugins/ menu. Go to *Image/Adjust/Brightness / Contrast* and press *Reset* in the B&C panel now displayed. Next zoom in on the image to a 400% zoom (see fig 23).

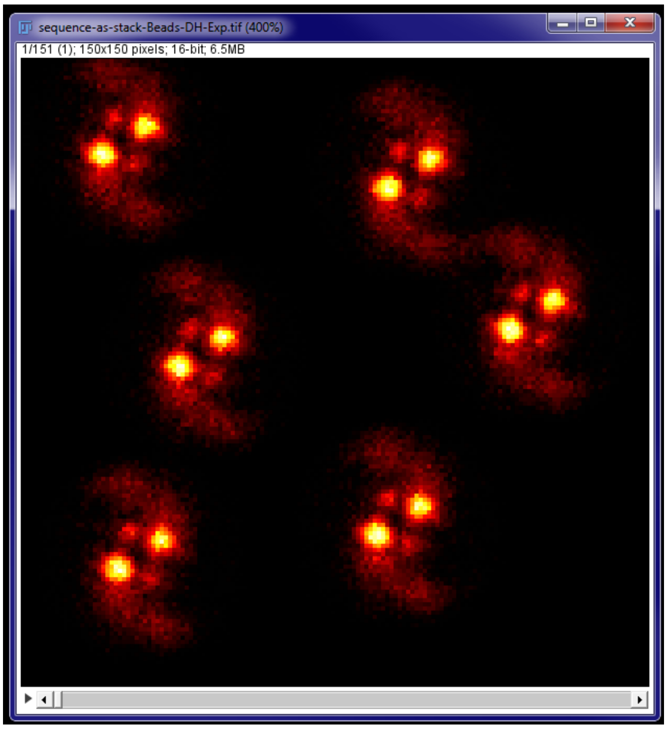

Figure 23 First slice of the calibration stack for Double Helix.

Proceed to select the correct modality in the dropdown menu (*Double Helix*) and press *Calibrate* (see fig 24).

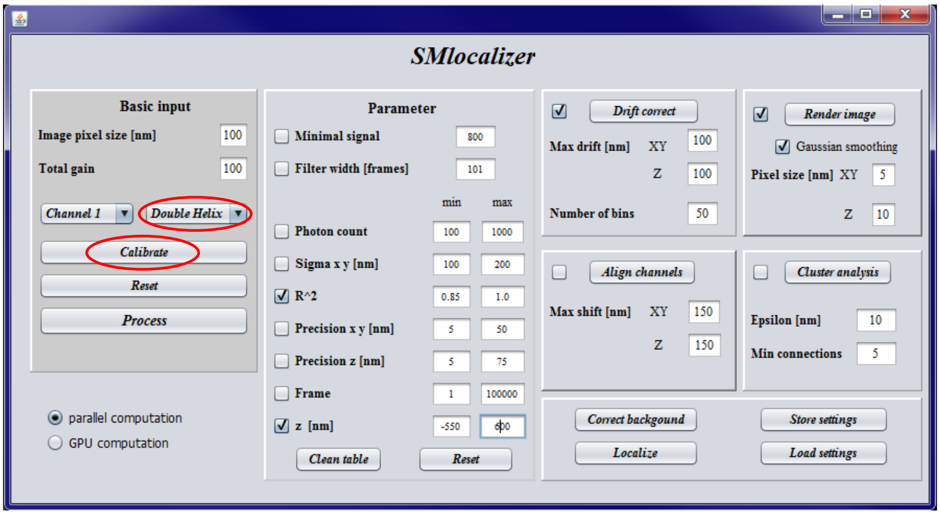

Figure 24 SMLocalizer GUI with relevant button and menu for calibration.

The software will proceed to ask the user for the z-step size for the stack provided (see fig 25, here 10 nm).

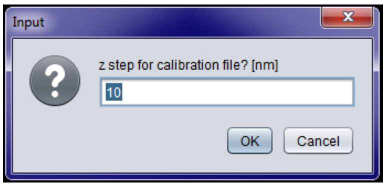

Figure 25 Z step size for calibration file.

The calibration algorithm will now find the optimal parameters for generating the calibration lookup table. Once complete a result calibration curve will be and an empty result table displayed (see fig 26).

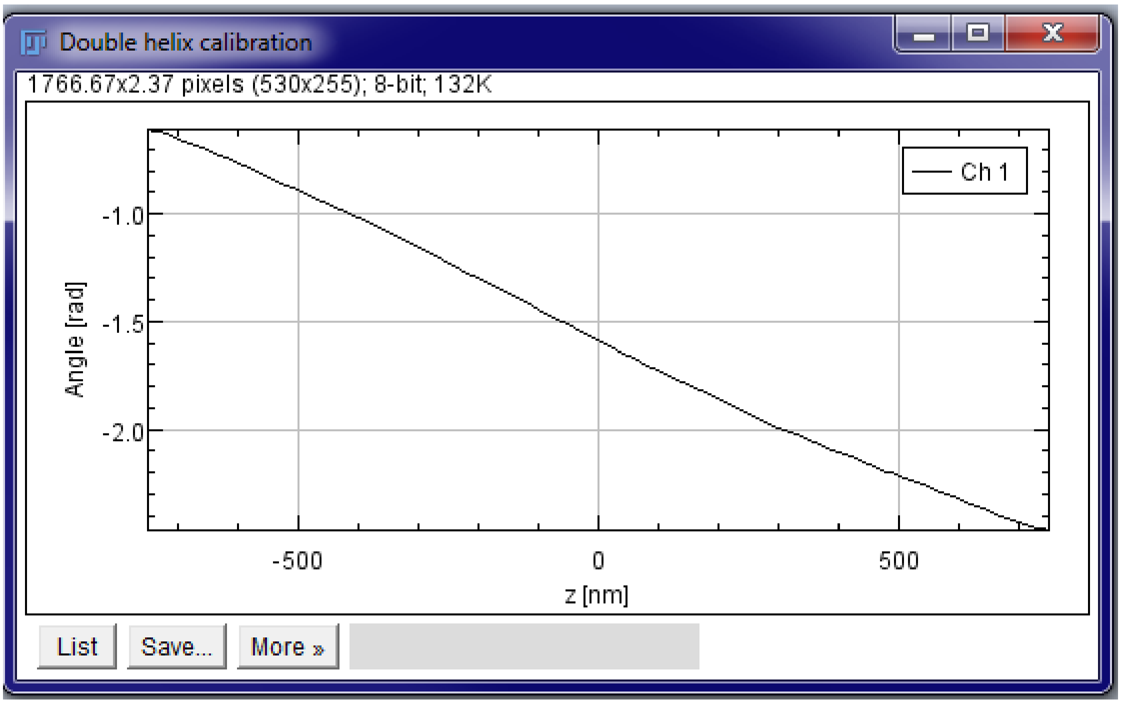

Figure 26 Calibration curve for Double Helix.

The calibration curve will be stored in ImageJ for future use. The next step is to close all open windows and load the experimental file (*sequence-as-stack-MT0.N1.LD-DH-Exp*).

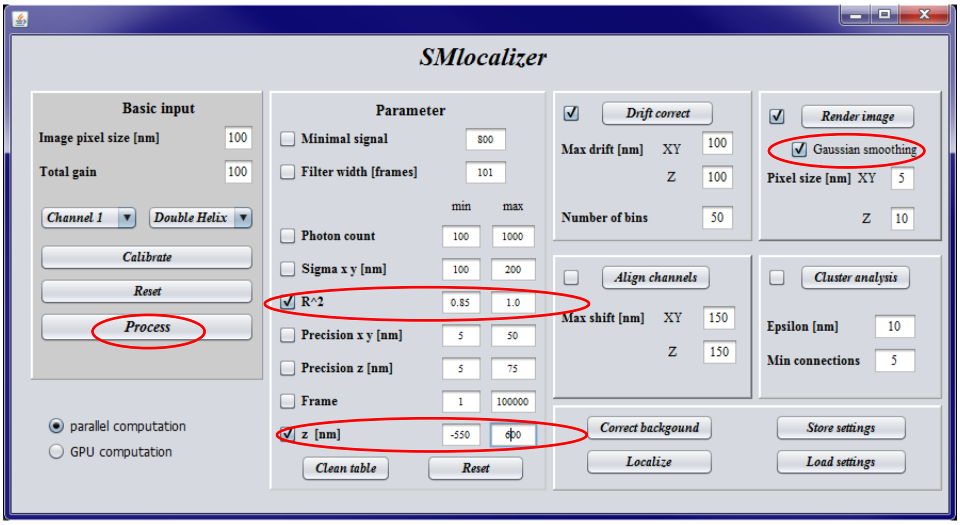

Figure 27 GUI for 3D double helix fitting.

See figure 27 for settings chosen for double helix fitting. Change the values for *z* limits to include -550 through 600 nm and activate *z* as parameter for selection. The data does not contain sufficient particles for drift correction (and does indeed not include drift) so drift correction can either be left on or be turned off (see fig 28 for output if left checked). Finally check that the image modality is set to *Double Helix*. Once all changes have been made, press *Process*. Alternatively press *Correct background*, once complete press *Localize* and once that is complete finally press *Render image*.

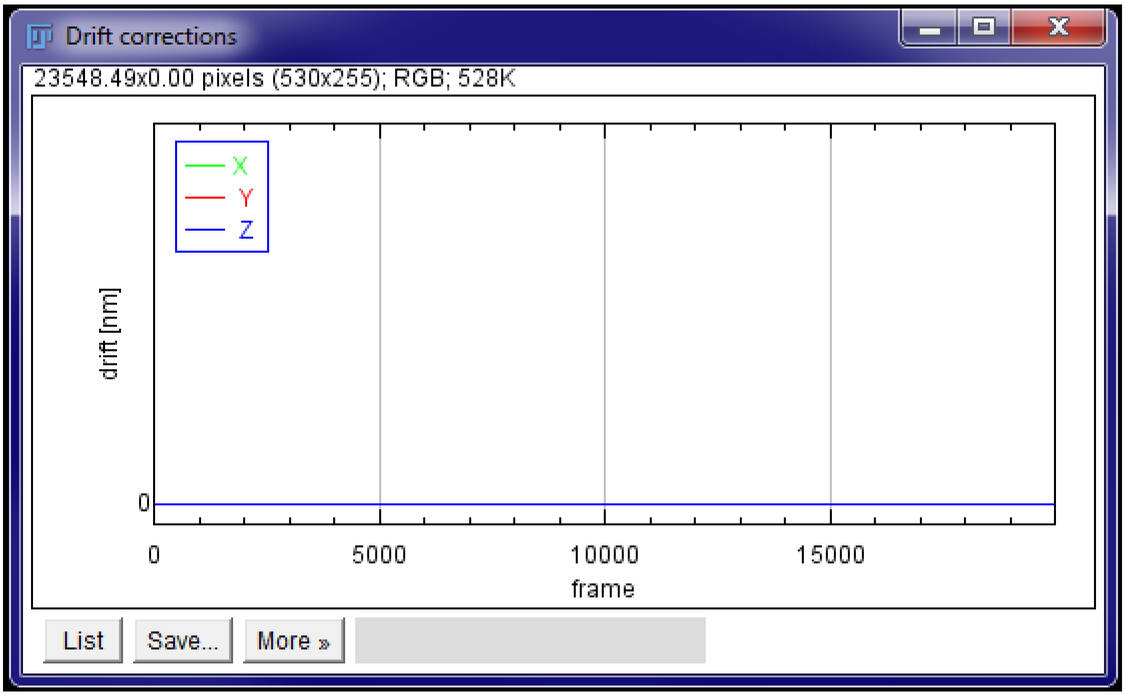

Figure 28 If drift correction was active the following window will appear, showing that no drift compensation was applied.

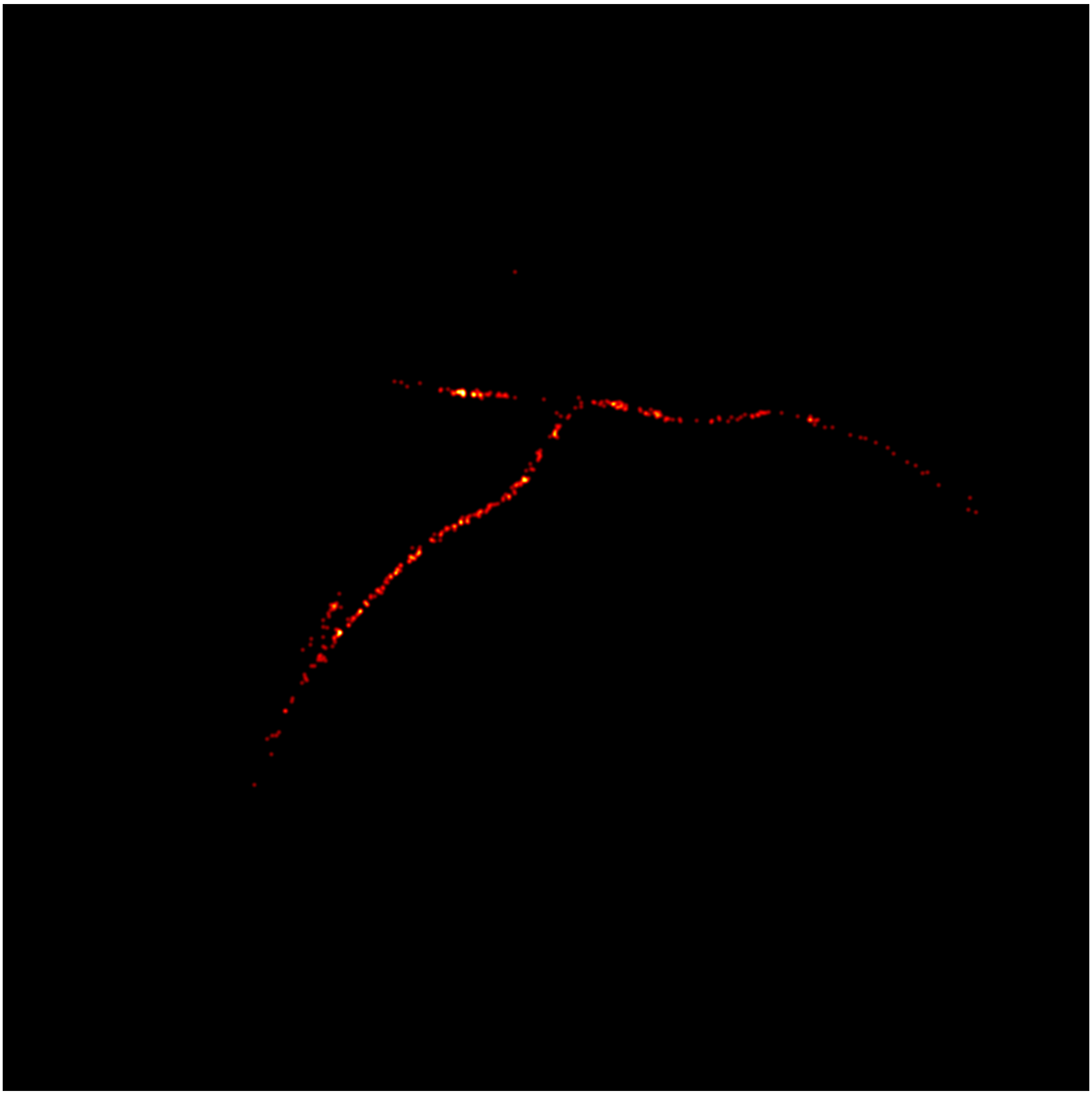

Figure 29 Slice 67 from the produced image stack after the histogram has been reset and lookup table has been modified to Red Hot.

Conclude the rendering by color depth coding the image. Select the image stack and go to one of the denser slices, in this example 67 (see fig 29 for a view of this slice). Go to *Image/Adjust/Brightness / Contrast* and press *Reset* in the B&C panel now displayed. Following this, go to *Image/Hyperstacks/Temporal-Color code*, select *Thermal* as lookup table, and press *Go* (see fig 30 for output).

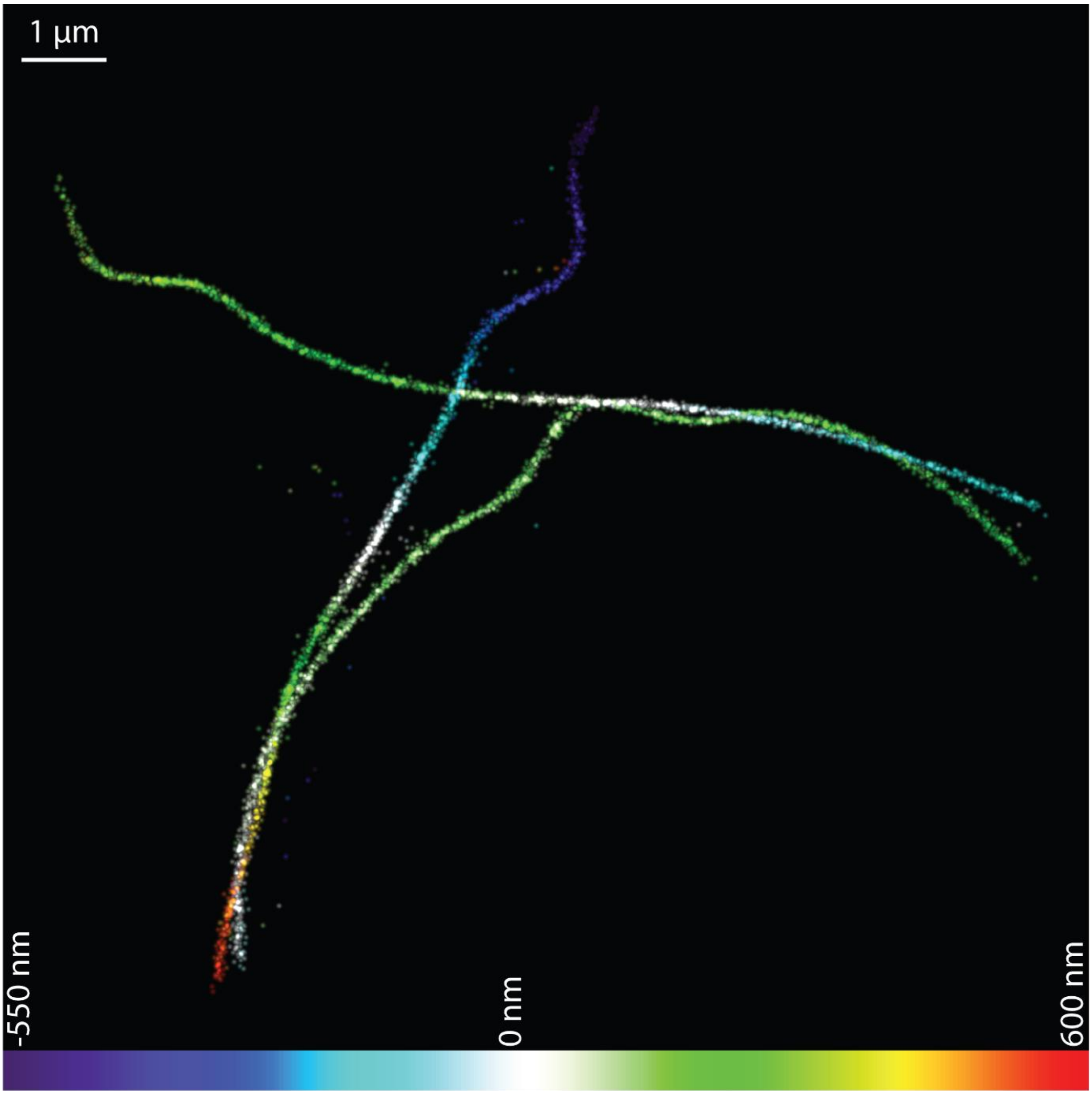

Figure 30 Rendering of results from fitting the double helix 3D dataset.

4.3 MITOFILIN PRILM

Provided on https://sourceforge.net/projects/smlocalizer/ are three datasets that are used in this tutorial. A bead stack for PRILM calibration, an overview widefield image and a PRILM modality dataset (see fig 31).

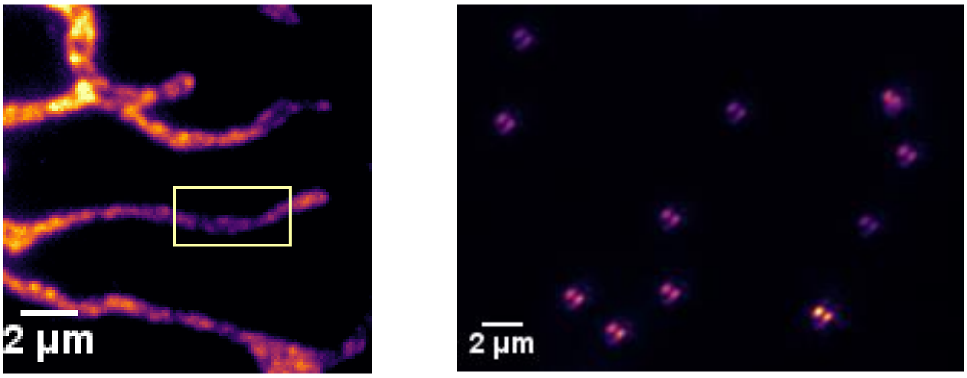

Figure 31 WF image with cropped region marked for the PRILM dataset and central slice (101) from the PRILM calibration stack available on https://sourceforge.net/projects/smlocalizer/

The sample is a U2OS cell, fixed using paraformaldehyde and labeled with a primary antibody against Mitofilin[9] (proteintech, id: 10179-1-AP) and a secondary antibody labeled with Alexa Fluor 647. The images are acquired on a Carl Zeiss Elyra PS.1 using 642 nm activation and increasing back-pumping with 405 nm. A Plan-Apochromate 100x/1.46 Oil (Zeiss) objective was used and emission was collected through a 655 nm long pass filter. Pixel size was 100 x 100 nm, integration time was 25 ms and the gain on the EMCCD camera was set to 100.

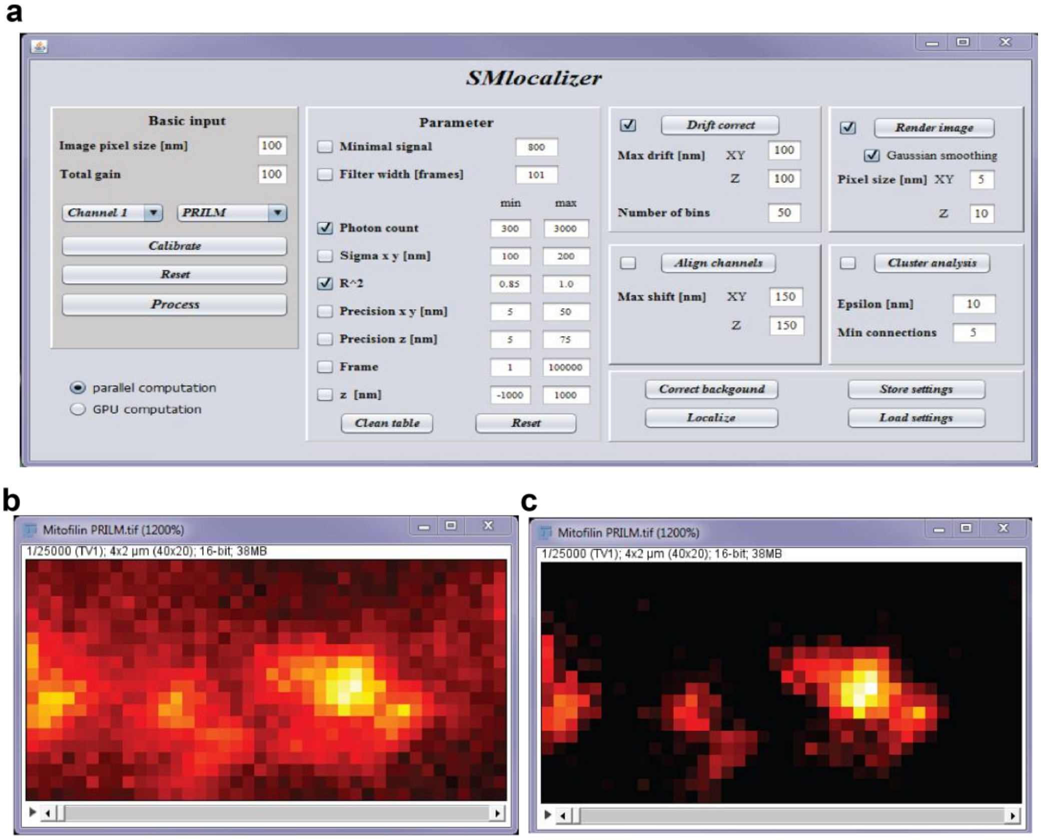

Figure 32 a) SMLocalizer GUI set for background correction of Mitofilin PRILM stack. b) Raw first frame of Mitofilin PRILM.tif zoomed in to 1200%. c) Background corrected first frame of Mitofilin PRILM.tif tif zoomed in to 1200% (after B&C was set to auto).

Load the *PRILM calibration stack* into ImageJ and start SMLocalizer. Select PRILM as image modality, set *Image pixel size* to 100 and press *Calibrate*. When asked, set *z-step* to 10 nm and press *ok.* Once completed a plot with the resulting angle-z graph will be displayed along with an empty results table. Close both and proceed to load the *Mitofilin PRILM* dataset into ImageJ (see fig 32).

With this information, the basic settings in SMLocalizer can be set (*Image pixel size*: 100, *Total gain*: 100). Input these settings into SMLocalizer and press *Process*. Once a results table has been displayed, it is time to change the parameter ranges. Select *Photon count* and set it to 300-3000 and change the *R^2* range to 0.7 – 1.0. PRILM distorts the PSF and a great fit against a Gaussian will not work against the more distorted (far from focus) events. There are too few particles to do any reasonable drift corrections with so proceed to change *Pixel size* to 5 and 5 nm (in Render image section) and proceed to press *Render Image* (see fig 33a). Do not have the *Gaussian smoothing* checkbox ticked. Increase the pixel values for the image by the command *Process/Math/Multiply* and set the value to 5000 and press ok (see fig 33b&c).

For final image rendering, four new images will be generated, all using the just generated 4000x2000x725nm tif stack:

1. Start with the command *Image/Stack/Reslice [/]…* and press ok. This will generate a new stack with the dimensions 4000x725 nm and show the x-z distribution. Proceed to use the command *Image/Stack/Z project* and select *Sum Slices* and press ok. Use the command *Process/Filters/Gaussian Blur* and set the radius to 2.0 and press OK. Use the command Image/Lookup tables/mpl-inferno and finish by setting the histogram range (*Image/Adjust/Brightness and Contrast*) using auto (see fig 33d).
2. Start with the command *Image/Stack/Reslice [/]…*, set *Start at* to *Left* and press ok. This will generate a new stack with the dimensions 2000x725 nm and show the y-z distribution. Proceed to use the command *Image/Stack/Z project* and select *Sum Slices* and press ok. Use the command *Process/Filters/Gaussian Blur* and set the radius to 2.0 and press OK. Use the command Image/Lookup tables/mpl-inferno and finish by setting the histogram range (*Image/Adjust/Brightness and Contrast*) using auto (see fig 33e).
3. Start with the command *Image/Stack/Z project* and select *Sum Slices* and press ok. Use the command *Process/Filters/Gaussian Blur* and set the radius to 2.0 and press OK. Use the command Image/Lookup tables/mpl-inferno and finish by setting the histogram range (*Image/Adjust/Brightness and Contrast*) using auto (see fig 33f).
4. Start with the command Process/Filters/Gaussian Blur 3D and set all values to two. Press ok and proceed with finding the highest intensity frame (# 59) and press Auto in the B&C window. Proceed with the command *Image/Hyperstacks/Temporal-Color* code, select *Thermal* in the dropdown menu, and press ok. The resulting image will be color coded for depth according to the generated *color time scale* window. White is in focus, blue shifted colder colors are below the focus and red shifted warmer colors are above the focus. (see fig 33g&h)

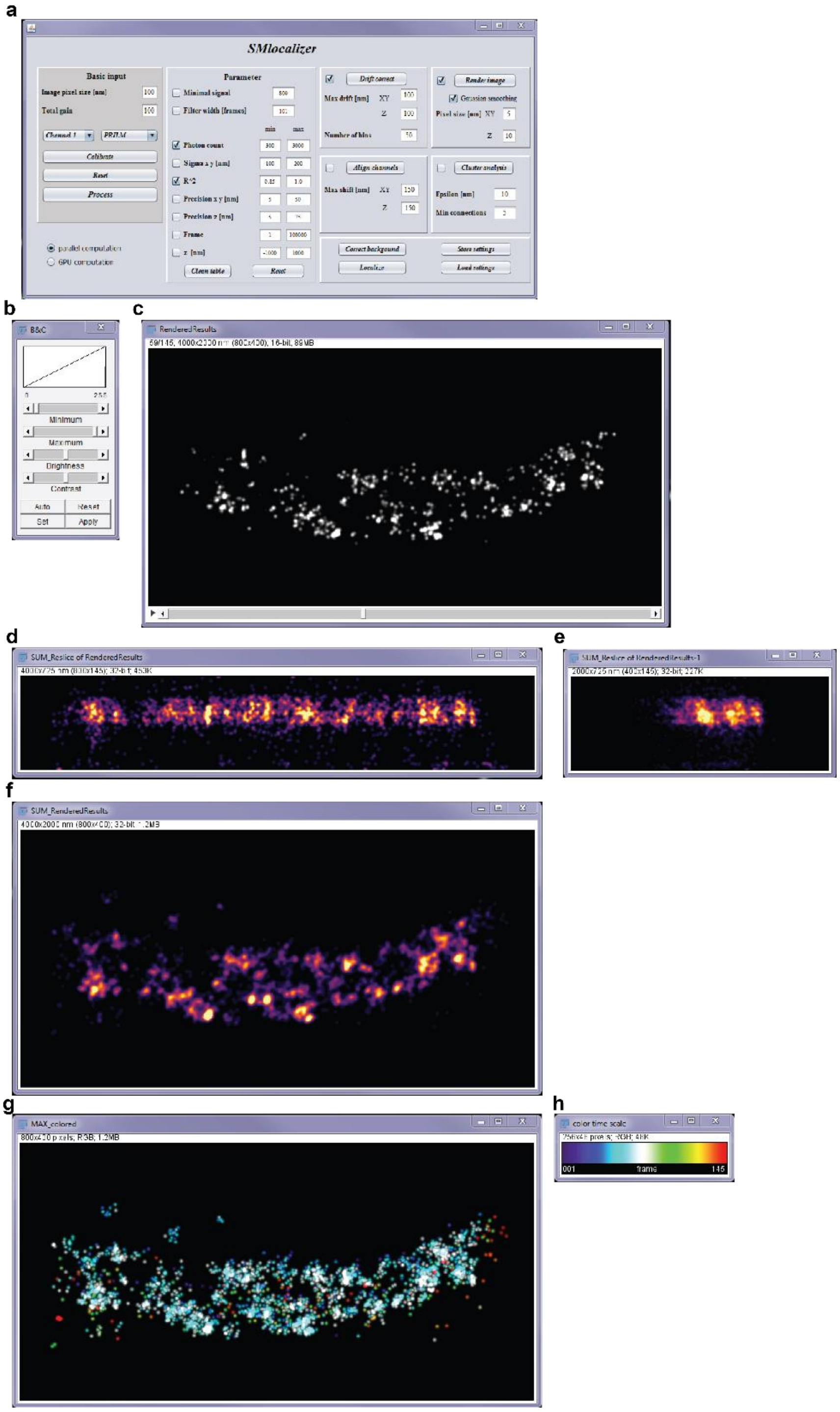

Figure 33 a) SMLocalizer GUI for image rendering. b) B&C window used for histogram changes. c) Slice 59 after "auto" has been pressed in B&C window. d) x-z projection of the sample. e) y-z projection of the sample. f) x-y projection of the sample. g) Color coded projection of the sample. h) Depth scale bar for g).

4.4 2D Gold bead tutorial – Drift correction

Provided on https://sourceforge.net/projects/smlocalizer/ is a bead sample dataset, *50nm_Gold_bead.tif*. Download the file for use in this tutorial, load it into ImageJ and start SMLocalizer (see fig 34a&b).

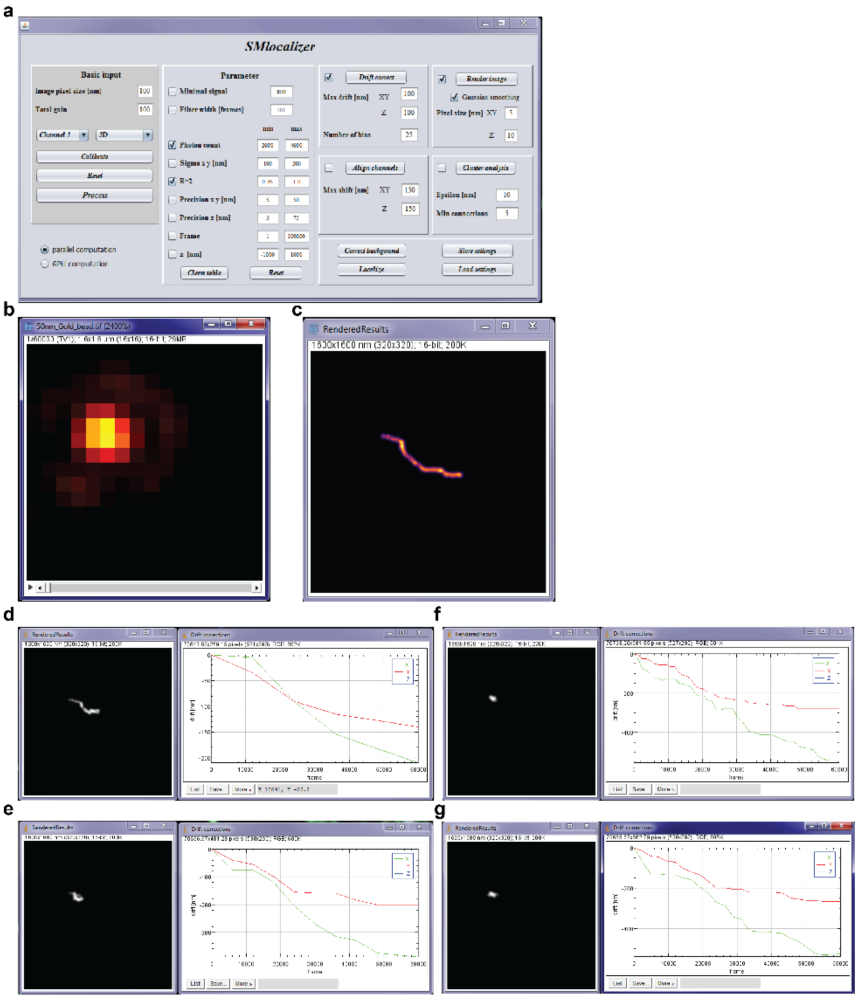

Figure 34 a) SMLocalizer GUI with all settings correct for this tutorial. b) Raw first frame of the input data zoomed in to 2400%. c) Initial, non corrected, output showing the introduced drift in of the bead with mpl-inferno lookup table. d) Drift correction in x and y calculated by SMLocalizer for 5 bins. e) Drift correction in x and y calculated by SMLocalizer for 10 bins. f) Drift correction in x and y calculated by SMLocalizer for 25 bins. g) Drift correction in x and y calculated by SMLocalizer for 50 bins.

As this is a bead sample, we can not background correct this image stack as that would remove the non-blinking bead from our dataset. Instead we directly localize the particles by hitting the *Localize* key. Wait for a results table to be displayed, change the *Photon range* to 2000 – 4000, tick the checkbox for *Gaussian smoothing* and press *Render image* to display the non drift corrected results (see fig 34c). Next we calculate and apply the drift correction for this dataset. Press *Drift correct* and wait for the Drift corrections plot to appear. Conclude with pressing *Render image again*. In the figure 34 d-g above we used 4 different values for number of bins, both under and oversampling.

5 ARCHITECTURE AND ALGORITHMS

5.1 BACKGROUND CORRECTION

5.1.1 Static events removal

SMLocalizer performs background correction based on the work of Hoogendoorn el al[3]. In short, each pixel is median filtered in time through the following steps:

1. The mean intensity in each frame is calculated.
2. Pixels are normalized to the frame mean.
3. The *filter width* number of pixels is used to calculate the running median for a specific pixel.
4. The median is subtracted from the original normalized pixel value and is subsequently rescaled using the frame mean.
5. The method removes static (over at least half the *filter width*) objects and retains transient increases (blinking events).

Pseudo code for performing the background correction (see Hoogendoorn el al[3] for details):

for (all frames)

$I_t = <I_{x,y,t}>_{x,y}$

$N_{x,y,t} = I_{x,y,t}/I_t$

for (all frames)

$I_{x,y,t} = I_{x,y,t} - I_t \cdot median\{ N_{x,y,t-w,\ldots} N_{x,y,t}, \ldots N_{x,y,t+w} \}$

$I_{x,y,t}$ *: pixel intensity*

I_t *: frame mean intensity*

$N_{x,y,t}$ *: Normalized pixel intensity*

Best results are obtained when the filter window width is > 10x the duration of blinking events.

5.1.2 Shot noise

Following median filtering described above each frame is run through a bicubic B-spline filter to further clean out the signal[10]. Each pixel is processed with the following 5x5 kernel:

0.0015257568…, 0.0036617187…, 0.0286859863…, 0.0036617187…, 0.0015257568…
0.0036617187…, 0.0087878906…, 0.0688445311…, 0.0087878906…, 0.0036617187…,
0.0286859863…, 0.0688445311…, 0.5393295900…, 0.0688445311…, 0.0286859863…,
0.0036617187…, 0.0087878906…, 0.0688445311…, 0.0087878906…, 0.0036617187…,
0.0015257568…, 0.0036617187…, 0.0286859863…, 0.0036617187…, 0.0015257568…

5.2 FITTING

5.2.1 2D fitting

Gaussian fitting is done iteratively with decreasing step size. The 2D elliptic tilted Gaussian description used is:

$$\theta_A = \frac{\cos^2(\vartheta)}{2\sigma_x^2} + \frac{\sin^2(\vartheta)}{2\sigma_y^2}$$

$$\theta_B = -\frac{\sin(2\vartheta)}{4\sigma_x^2} + \frac{\sin(2\vartheta)}{4\sigma_y^2}$$

$$\theta_C = \frac{\cos^2(\vartheta)}{2\sigma_x^2} + \frac{\sin^2(\vartheta)}{2\sigma_y^2}$$

$$f(x,y) = amp \cdot e^{-[\theta_A \cdot (x-x_0)^2 + \theta_B \cdot (x-x_0)\cdot(y-y_0) + \theta_C \cdot (x-x_0)^2]} - b$$

Where *b* is the background and *amp* the amplitude of the function. The residual error of fitting is calculated as:

$$R^2 = 1 - \frac{SSE}{SST}$$

$$SSE = \sum_{x,y} (f(x,y) - \bar{y})^2$$

$$SST = \sum_{x,y} (y(x,y) - \bar{y})^2$$

Where y is the measured pixel values and $\bar{y}$ is the mean value.

Optimization is performed by minimizing SSE/SST after initial guesses of input parameters has been done.

- x_0 and y_0 are estimated as the weighted centroid.
- σ_x and σ_y are evaluated between 80 nm and 200 nm and the combination yielding the best fit is used for initial estimate.
- *amp* is estimated as the central pixel intensity.
- *b* is estimated to 0.
- ϑ is estimated as 0.

```
for (all regions)
   for (σ_x = 80:200 && σ_y = 80:200)
    find((σ_x , σ_y ) → min(G(P))

   while(optimize)
          P(current) += stepSize(current)
          if(P(current) within bounds)
             if(G(P) > G(P_old)) // if we did not improve
                 if (stepSize(current > 0)
                    stepSize(current) *= -1   // change direction
                 else
                     stepSize(current) *= -1.5   // change direction and decrease
           else // if outside bounds
                 if (stepSize(current > 0)
                    stepSize(current) *= -1   // change direction
                 else
                     stepSize(current) *= -1.5   // change direction and decrease
            if (loops = max loops || G(P) – G(P_old) < convergence)
                      optimize = false
            else
                          current = next // evaluate next parameter.
```

G(P) and G(P_{old}) evaluates the Gaussian with current or last rounds parameters and returns the SSE/SST (see above).

5.2.2 3D fitting

All 3D modalities start with 2D fitting using parameter settings obtained from the calibration file. The fitted particles are then translated using a lookup table and raw data to obtain 3D information. For all 3D modalities as part of the calibration, the x-y offset from the frame in focus is determined as function of z. This correction is applied to the final x-y coordinates.

5.2.2.1 PRILM

PRILM fitting starts with generation of a calibration lookup table from an image stack of beads.

```
for (gaussianWindow = 500 nm:900 nm)
    for (intensityMinimum = 0.5:0.7)
        for (lobeDistance = 600 nm:1000 nm)
            set minimum intensity for peak recognition by intensityMinimum*[central frame max intensity]
            Fit all located particles as in 2D (see above)
            Remove fits with r_square < 0.70
            Set particle z height to frameNumber*z-stepSize (from user)
            for (all particles with include==1)
                set Particle(current).include = 2
                find (Particle(all).include==1 closest to Particle(current))
                if (distance(Particle(closest)-Particle(current)) < lobeDistance)
                    Particle(closest).include = 2 (exclude from further searches)
                    dx = Particle(current).x0 – Particle(closest).x0
                    dy = Particle(current).y0 – Particle(closest).y0
                    angle = atan2(dy,dx)
                Sum up angle for all particles from a given frame and calculate the mean angle, add to angle[z]
            end
            calculate length of angle[z] which is unambiguous.
        end
    end
end
take the longest angle[z], store it and store gaussianWindow and lobeDistance that yielded this.
Calculate x-y-z channel offset from channel 1 to all other channels and store it.
```

Once a calibration lookup table has been generated 3D fitting can be performed. The window used for fitting and the maximum distance allowed between lobes are taken from the calibration file, as is the chromatic corrections.

```
Fit all objects using 2D algorithms and user provided minimum intensity.
for (Particle(all).include == 1)
    set Particle(current).include = 2
    find within same frame and channel distance(Particle(current),Particle(Particle with include == 1)) < lobeDistance
    Particle(closest).include = 2
    dx = Particle(current).x0 – Particle(closest).x0
    dy = Particle(current).y0 – Particle(closest).y0
    angle = atan2(dy,dx)
    newParticle.x0 = (Particle(current).x0 + Particle(closest).x0)/2 + xCorrect(newParticle.z0,channel)
    newParticle.y0 = (Particle(current).y0 + Particle(closest).y0)/2 + yCorrect(newParticle.z0,channel)
    newParticle.z0 = angleCalibrationLookUpTable[angle]
    newParticle.sigma_x = (Particle(current). sigma_x + Particle(closest). sigma_x)/2
    newParticle.sigma_y = (Particle(current). sigma_y + Particle(closest). sigma_y)/2
    newParticle.photons = Particle(current). photons + Particle(closest). Photons
    newParticle.precision_x = 0.5(Particle(current). precision_x + Particle(closest). precision_x)
    newParticle.precision_y = 0.5(Particle(current). precision_y + Particle(closest). precision_y)
    newParticle.precision_z = error propagation, see 5.2.3
    newParticle.r_square = min(Particle(current). r_square + Particle(closest). r_square)
    if (Particle(current).channel > 1) shift x-y-z by calibrated offset.
end
```

5.2.2.2 Biplane

Biplane fitting starts with generation of a calibration lookup table from an image stack of beads.

```
for(all frames)
  pixelvalue = pixelvalue – frameMedian
end
for (gaussianWindow = 500 nm:900 nm)
   for (intensityMinimum = 0.4:0.7)
        for (max2Dsigma = 200 nm:350 nm)
             set minimum intensity for peak recognition by intensityMinimum*[central frame max intensity]
             Fit all located particles as in 2D (see above)
             Remove fits with r_square < 0.80
            Set particle z height to frameNumber*z-stepSize (from user)
            for (all particles with frame within 5 of center)
                 calculate mean offset in x-y
            end
            for (all particles)
                  check for second fitted particle offset away, use best fitted particle for x-y if found
                  sum up the pixels surrounding the center pixel at particle and offset away (gaussianWindow wide)
                  ratio[z] = intensity_left / intensity_right
                  calculate mean ratio[z] for each frame and channel
            end
            calculate length of ratio[z] which is unambiguous.
        end
   end
end
take the longest ratio[z], store it and store gaussianWindow and max2Dsigma that yielded this.
Calculate x-y-z channel offset from channel 1 to all other channels and store it.
```

Once a calibration lookup table has been generated 3D fitting can be performed. The window used for fitting and the maximum distance allowed between lobes are taken from the calibration file, as is the chromatic corrections.

```
Fit all objects using 2D algorithms and user provided minimum intensity.
for (Particle(all).include == 1)
    find (Particle offset away), use best fit for x-y
    intensityRatio = sum up pixelvalues gaussian fit window surrounding center pixel at center and offset away.
    newParticle.x0 = Particle(bestFit).x0 + xCorrect(newParticle.z0,channel)
    newParticle.y0 = Particle(bestFit).y0 + yCorrect(newParticle.z0,channel)
    newParticle.z0 = ratioCalibrationLookUpTable[intensityRatio]
    newParticle.sigma_x = Particle(bestFit). sigma_x
    newParticle.sigma_y = Particle(bestFit). sigma_y
    newParticle.photons = summed intensities surrounding both centras.
    newParticle.precision_x = Particle(bestFit). precision_x
    newParticle.precision_y = Particle(bestFit). precision_y
    newParticle.precision_z = 600 / sqrt(newParticle.photons)
    newParticle.r_square =Particle(bestFit). r_square
   if (Particle(current).channel > 1) shift x-y-z by calibrated offset.
end
```

5.2.2.3 Double helix

Double helix fitting starts with generation of a calibration lookup table from an image stack of beads.

```
for (gaussianWindow = 500 nm:900 nm)
   for (intensityMinimum = 0.5:0.7)
       for (lobeDistance = 600 nm:1000 nm)
           set minimum intensity for peak recognition by intensityMinimum*[central frame max intensity]
           Fit all located particles as in 2D (see above)
           Remove fits with r_square < 0.80
          Set particle z height to frameNumber*z-stepSize (from user)
          for (all particles with include==1)
                 set Particle(current).include = 2
               find (Particle(all).include==1 closest to Particle(current))
               if (distance(Particle(closest)-Particle(current)) < lobeDistance)
                   Particle(closest).include = 2 (exclude from further searches)
                     dx = Particle(current).x0 – Particle(closest).x0
                    dy = Particle(current).y0 – Particle(closest).y0
                    angle = atan2(dy,dx)
                 Sum up angle for all particles from a given frame and calculate the mean angle, add to angle[z]
          end
          calculate length of angle[z] which is unambiguous.
      end
   end
end
take the longest angle[z], store it and store gaussianWindow and lobeDistance that yielded this.
Calculate x-y-z channel offset from channel 1 to all other channels and store it.
```

Once a calibration lookup table has been generated 3D fitting can be performed. The window used for fitting and the maximum distance allowed between lobes are taken from the calibration file, as is the chromatic corrections.

```
Fit all objects using 2D algorithms and user provided minimum intensity.
for (Particle(all).include == 1)
     set Particle(current).include = 2
     find within same frame and channel distance(Particle(current),Particle(Particle with include == 1)) < lobeDistance
    Particle(closest).include = 2
    dx = Particle(current).x0 – Particle(closest).x0
    dy = Particle(current).y0 – Particle(closest).y0
    angle = atan2(dy,dx)
   newParticle.x0 = (Particle(current).x0 + Particle(closest).x0)/2 + xCorrect(newParticle.z0,channel)
   newParticle.y0 = (Particle(current).y0 + Particle(closest).y0)/2 + yCorrect(newParticle.z0,channel)
   newParticle.z0 = angleCalibrationLookUpTable[angle]
   newParticle.sigma_x = (Particle(current). sigma_x + Particle(closest). sigma_x)/2
   newParticle.sigma_y = (Particle(current). sigma_y + Particle(closest). sigma_y)/2
   newParticle.photons = Particle(current). photons + Particle(closest). Photons
   newParticle.precision_x = 0.5(Particle(current). precision_x + Particle(closest). precision_x)
   newParticle.precision_y = 0.5(Particle(current). precision_y + Particle(closest). precision_y)
   newParticle.precision_z = error propagation, see 5.2.3
   newParticle.r_square = min(Particle(current). r_square + Particle(closest). r_square)
  if (Particle(current).channel > 1) shift x-y-z by calibrated offset.
end
```

5.2.2.4 Astigmatism

Astigmatism fitting starts with generation of a calibration lookup table from an image stack of beads.

```
for (gaussianWindow = 1500 nm:1900 nm)
   for (intensityMinimum = 0.5:0.7)
        for (max2Dsigma = 400 nm:800 nm)
             set minimum intensity for peak recognition by intensityMinimum*[central frame max intensity]
             Fit all located particles as in 2D (see above)
             Remove fits with r_square < 0.80
            Set particle z height to frameNumber*z-stepSize (from user)
            for (all particles)
                 ratio[z] = sigma_x / sigma_y
                 maxDim[z] = max(sigma_x,sigma_y)
            end
            calculate length of ratio[z] which is unambiguous with help of maxDim[z] by finding maximum maxDim giving this
        end
   end
end
take the longest ratio[z], store it and store gaussianWindow,maxDim and max2Dsigma that yielded this.
Calculate x-y-z channel offset from channel 1 to all other channels and store it.
```

Once a calibration lookup table has been generated 3D fitting can be performed. The window used for fitting and the maximum distance allowed between lobes are taken from the calibration file, as is the chromatic corrections.

```
Fit all objects using 2D algorithms and user provided minimum intensity.
for (Particle(all).include == 1)
    ratio = sigma_x/sigma_y
    if (max(sigma_x,sigma_y)< maxDim)
       newParticle.x0 = Particle(current).x0 + xCorrect(newParticle.z0,channel)
       newParticle.y0 = Particle(current).y0 + yCorrect(newParticle.z0,channel)
       newParticle.z0 = ratioCalibrationLookUpTable[intensityRatio]
       newParticle.sigma_x = min(Particle(current). sigma_x, Particle(current). sigma_y)
       newParticle.sigma_y = min(Particle(current). sigma_x, Particle(current). sigma_y)
       newParticle.photons = summed intensities surrounding both centras.
       newParticle.precision_x = min(Particle(current). precision_x, Particle(current). precision_y)
       newParticle.precision_y = min(Particle(current). precision_x, Particle(current). precision_y)
       newParticle.precision_z = 600 / sqrt(newParticle.photons)
       newParticle.r_square =Particle(current). r_square
       if (Particle(current).channel > 1) shift x-y-z by calibrated offset.
   end
end
```

5.2.3 Z precision estimate for PRILM and double helix

The error in x-y localization propagates to an error in z determination. By shifting the centers for the two lobes by their precision in x and y and calculating a new angle we obtain the uncertainty in angle determination for a given double psf localization. By calculating the resulting z value we get boundaries for the uncertainty of z determination for the given localization. In figure 35a this shift and subsequent angle calculation is demonstrated. In figure 35b the angle is interpreted for a double helix calibration curve.

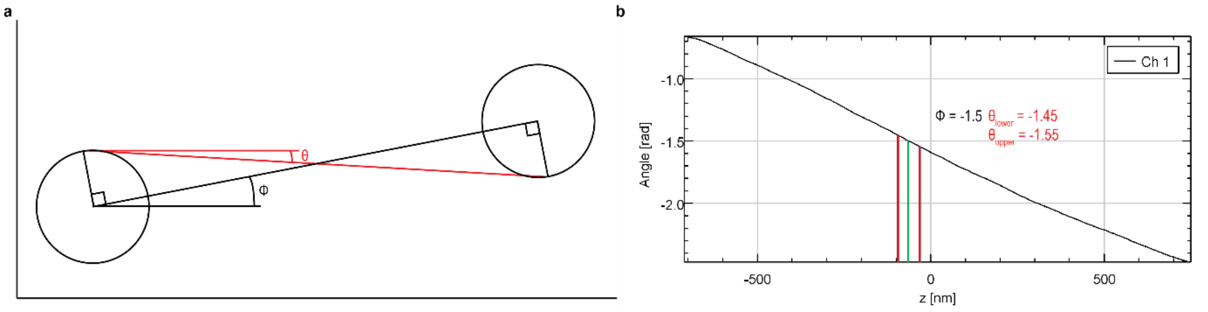

Figure 35. A shows the two centers of the lobes from a PRILM or double helix psf with circles showing the precision in x-y. Moving along the normal to the vector connecting the two centers to this border two new points are obtained. The angle of the vector connecting these two new points are then calculate and the difference from the original angle saved. The second bound is obtained by reversing the direction of travel along the normal symmetry tells us that this second angle difference to original will be the same as the first one.

If either the upper or lower border for the precision determination falls outside the calibration curve a value of 1000 will be reported. If both upper and lower bound falls within the calibration curve the mean z offset will be reported as z precision.

5.3 DRIFT CORRECTION AND CHANNEL ALIGNMENT

Drift correction is performed by calculating the cross correlation between different bins of particles (in time). For each bin an image (10x10x10 nm) (2D or 3D) is generated. The maximum correlation between two adjacent bins is obtained and the shift applied to the second bins data points.

For each bin the following pseudocode is executed:

$$xCorr(d) = \frac{\sum(\boldsymbol{x_i}-\overline{x})(\boldsymbol{y_{i-d}}-\overline{y})}{\sqrt{\sum(\boldsymbol{x_i}-\overline{x})^2}\sqrt{(\boldsymbol{y_{i-d}}-\overline{y})^2}}$$

where $\mathbf{x}$, $\mathbf{y}$ *are 3 dimensional arrays,* $\mathbf{i}$ *is a 3 dimensional index,* $\mathbf{d}$ *is a 3 dimensional shift.* $\overline{x}$ *and* $\overline{y}$ *represent array means. Non overlapping voxels due to shift are discarded.*
output = find shift →max (xCorr)

5.4 IMAGE RENDERING

For single channel and multichannel a single image (with multiple slices for multichannel data) is generated. Particle coordinates are rounded to nearest multiple of pixel size and the resulting pixel coordinate value is increased by 1.

If *Gaussian smoothing* is selected the image values will be multiplied by 1000 and a Gaussian filter with 2-pixel filter radius will be applied to the image.

All images are calibrated to get accurate dimensions.

5.5 CLUSTER ANALYSIS

Cluster analysis will perform cluster analysis on the particles within a given channel that has parameters within a user set ok range. The current version implements DBSCAN for 2D clusters using the apache commons framework. See http://commons.apache.org/proper/commons-math/apidocs/org/apache/commons/math4/ml/clustering/DBSCANClusterer.html for details on the functions called where *eps* = *Epsilon* and *minPts* = *Minimum connections*. The results are given as a new rendered image with only the clusters found present and an update to the result list with a column for cluster id.

6 REFERENCES

1 Schindelin, J., Rueden, C. T., Hiner, M. C. & Eliceiri, K. W. The ImageJ ecosystem: An open platform for biomedical image analysis. *Molecular Reproduction and Development* **82**, 518-529, doi:10.1002/mrd.22489 (2015).

2 Schindelin, J. *et al.* Fiji: an open-source platform for biological-image analysis. *Nat Meth* **9**, 676-682, doi:http://www.nature.com/nmeth/journal/v9/n7/abs/nmeth.2019.html#supplementary-information (2012).

3 Hoogendoorn, E. *et al.* The fidelity of stochastic single-molecule super-resolution reconstructions critically depends upon robust background estimation. *Scientific Reports* **4**, 3854, doi:10.1038/srep03854

http://www.nature.com/articles/srep03854#supplementary-information (2014).

4 Baddeley, D., Cannell, M. B. & Soeller, C. Three-dimensional sub-100 nm super-resolution imaging of biological samples using a phase ramp in the objective pupil. *Nano Research* **4**, 589-598, doi:10.1007/s12274-011-0115-z (2011).

5 Juette, M. F. *et al.* Three-dimensional sub-100 nm resolution fluorescence microscopy of thick samples. *Nat Meth* **5**, 527-529, doi:http://www.nature.com/nmeth/journal/v5/n6/suppinfo/nmeth.1211_S1.html (2008).

6 Pavani, S. R. P. *et al.* Three-dimensional, single-molecule fluorescence imaging beyond the diffraction limit by using a double-helix point spread function. *Proceedings of the National Academy of Sciences* **106**, 2995-2999, doi:10.1073/pnas.0900245106 (2009).

7 Huang, B., Wang, W., Bates, M. & Zhuang, X. Three-Dimensional Super-Resolution Imaging by Stochastic Optical Reconstruction Microscopy. *Science* **319**, 810 (2008).

8 Sander, J., Ester, M., Kriegel, H.-P. & Xu, X. Density-Based Clustering in Spatial Databases: The Algorithm GDBSCAN and Its Applications. *Data Mining and Knowledge Discovery* **2**, 169-194, doi:10.1023/A:1009745219419 (1998).

9 Jans, D. C. *et al.* STED super-resolution microscopy reveals an array of MINOS clusters along human mitochondria. *Proceedings of the National Academy of Sciences* **110**, 8936-8941, doi:10.1073/pnas.1301820110 (2013).

10 Unser, M. A Perfect Fit for Signal and Image Processing. *IEEE Signal Processing Magazine* **16**, 22-38 (1999).
