# Supplementary material for: SMLocalizer, a GPU accelerated ImageJ plugin for single molecule localization microscopy
Source: Bioinformatics. 2017 Sep 4;34(1):137–8. doi: 10.1093/bioinformatics/btx553 (PMC5870682; doi:10.1093/bioinformatics/btx553)
Supplement: Supplementary Methods [file supplemental_methods_btx553.docx]

**PRILM test sample preparation and imaging**

Human Bone Osteosarcoma Epithelial (U2OS) cells were cultured on #1.5 18 mm-round coverslips (Marienfeld). Cells were washed in 37 ºC PBS (137 mM NaCl, 2.7 mM KCl, 10 mM Na2HPO4, 1.8 mM KH2PO4, 1 mM CaCl2, 0.5 mM MgCl2, pH 7.4, all chemicals from Sigma Aldrich) and subsequently fixed for 10 min using 4 % paraformaldehyde (Sigma Aldrich). Next cells were washed briefly and permabilized using 0.5 % Triton-X100 (Sigma Aldrich). Cells were washed once in iPBS and blocked for 10 min with 5 % filtered Bovine Serum Albumine (BSA, Sigma Aldrich). Cells were stained in room temperature for 60 min with a 1:200 dilution of Mitofilin antibody (Proteintech 10179-1-AP) in 5 % BSA. Cells were subsequently washed 3x5 min with iPBS and stained with secondary goat anti-mouse alexa fluor 647 (ThermoFischer Scientific) at 1:250 dilution in 5 % BSA. Cells were washed 5x5 min with iPBS and subsequently mounted and imaged. Samples were mounted on caveat slides (Marienfeld) with the following STORM buffer: 1:100 GLOX 100x (10 mg glucose oxidase in 180 μl of 10 mM Tris (pH 7.0) and 10 mM NaCl) and 1:100 1 M MEA (77 mg cystamine in 1 ml 1:12 HCl) in 50 mM Tris-Cl (pH 8.0) 10 mM NaCl and 10% glucose. Samples were sealed with Twinsil.

PRILM imaging was performed on a Carl Zeiss Elyra PS.1 microscope using 642 nm excitation laser and 405 nm activation laser. A Plan-Apochromate 100x/1.46 (Zeiss) objective was used and fluorescence was detected on a liquid cooled EMCCD camera (Andor Technology) with an optical PRILM element for PSF modulation in the beam path. Emission was collected through a 655 nm long pass filter with a integration time on the camera of 25 ms. A 12.8 x 12.8 μm area was imaged, with excitation laser set to 100 % (24.4 mW maximal input power) and activation laser linearly increasing from 0 to 0.5 % (100% = 10.1 mW maximal input power). Camera gain was set to 100 times and 25000 images were collected for each experiment.

**Ground truth synthetic datasets**

Ground truth synthetic datasets were generated as follows:

- A background free image (3200x3200 pixels, pixel size 1x1 nm) with a Gaussian point emitter (σ=150 nm) positioned slightly off-centre (x=1580,y=1610) was generated.
- The image was down sampled to 100x100 nm pixel size.
- A background noise stack (32x32x25000) was generated. Noise was generated for each pixel as a square random function with maximal value set to the desired peak signal to peak noise ratio.
- Randomly (p=0.1) the down sampled Gaussian image was added on top of the background noise images in sequence. The intensity of the Gaussian was adjusted using a Poisson distribution for a 1000 photon emitter.

Five datasets were generated with a peak signal to peak noise ratio ranging from 1:1 to 5:1. Each dataset was run through SMLocalizer, QuickPALM and ThunderSTORM and the resulting localization table was compared to ground truth. For each located event, the distance from ground truth centre was calculated. The mean error and the standard deviation of the error is included in Figure 1 a. The fraction of found false particles that were not introduced in the dataset that were found can be seen in Figure 1 b. The fraction of missed events can be found in Figure 1 c.
